# The efficacy of extracellular vesicles for acute lung injury in preclinical animal models: a meta-analysis

**DOI:** 10.1186/s12890-024-02910-4

**Published:** 2024-03-13

**Authors:** Xuefeng Zhang, Zongyong Cheng, Menghao Zeng, Zhihui He

**Affiliations:** 1 The Third Xiangya Hospital, Central South University, Changsha, Hunan China; 2 The Second Xiangya Hospital, Central South University, Changsha, Hunan China; 3 Department of Critical Care Medicine, the Third Xiangya Hospital, Central South University, Changsha, Hunan China; 4 138 Tongzibo Road, Yuelu District, Changsha, Hunan 410013 China

**Keywords:** Extracellular vesicles, Exosomes, Acute Lung injury, Acute respiratory distress syndrome, Meta-analysis

## Abstract

**Background:**

With the increasing research on extracellular vesicles (EVs), EVs have received widespread attention as biodiagnostic markers and therapeutic agents for a variety of diseases. Stem cell-derived EVs have also been recognized as a new viable therapy for acute lung injury (ALI) and acute respiratory distress syndrome (ARDS). To assess their efficacy, we conducted a meta-analysis of existing preclinical experimental animal models of EVs for ALI treatment.

**Methods:**

The database was systematically interrogated for pertinent data encompassing the period from January 2010 to April 2022 concerning interventions involving extracellular vesicles (EVs) in animal models of acute lung injury (ALI). The lung injury score was selected as the primary outcome measure for statistical analysis. Meta-analyses were executed utilizing RevMan 5.3 and State15.1 software tools.

**Results:**

The meta-analyses comprised 31 studies, exclusively involving animal models of acute lung injury (ALI), categorized into two cohorts based on the presence or absence of extracellular vesicle (EV) intervention. The statistical outcomes from these two study groups revealed a significant reduction in lung injury scores with the administration of stem and progenitor cell-derived EVs (SMD = -3.63, 95% CI [-4.97, -2.30], *P* < 0.05). Conversely, non-stem cell-derived EVs were associated with an elevation in lung injury scores (SMD = -4.34, 95% CI [3.04, 5.63], *P* < 0.05). EVs originating from stem and progenitor cells demonstrated mitigating effects on alveolar neutrophil infiltration, white blood cell counts, total cell counts in bronchoalveolar lavage fluid (BALF), lung wet-to-dry weight ratios (W/D), and total protein in BALF. Furthermore, pro-inflammatory mediators exhibited down-regulation, while anti-inflammatory mediators demonstrated up-regulation. Conversely, non-stem cell-derived EVs exacerbated lung injury.

**Conclusion:**

In preclinical animal models of acute lung injury (ALI), the administration of extracellular vesicles (EVs) originating from stem and progenitor cells demonstrably enhances pulmonary function. This ameliorative effect is attributed to the mitigation of pulmonary vascular permeability and the modulation of immune homeostasis, collectively impeding the progression of inflammation. In stark contrast, the utilization of EVs derived from non-stem progenitor cells exacerbates the extent of lung injury. These findings substantiate the potential utility of EVs as a novel therapeutic avenue for addressing acute lung injury.

## Introduction

ALI is a severe disease characterised by an excessive inflammatory response.ARDS is an acute inflammatory lung disease with more severe clinical manifestations than ALI, usually accompanied by increased alveolar permeability, causing severe alveolar oedema and further exacerbation of hypoxia, with high annual morbidity and mortality rates worldwide [1]. A worldwide observational investigation carried out from 2016 to 2017 across 145 Pediatric Intensive Care Units (PICUs) in 27 nations delineated a morbidity rate of 17% and a mortality rate of 3.2% among 23,280 patients [2]. Notwithstanding strides in therapeutic approaches, the absence of efficacious pharmaceutical interventions capable of substantially mitigating mortality and enhancing patient prognoses persists as a critical challenge [3, 4]. Currently, the diagnosis and treatment of a number of diseases, including stem cells and their related derivatives, have attracted the attention of researchers [5]. For example, a study has shown that serum EV-derived ASS1 levels predicted the progression of HEV-ALF and were strongly correlated with the severity of patients with HEV infection. In addition, serum levels of exosome-derived CPS1 have been reported as a diagnostic and prognostic biomarker for patients with HEV-ALF [6, 7]. Among various cell therapies, mesenchymal stem cells (MSCs) have been considered as a potential therapy for ALI/ARDS, and over the past decades, MSCs have played an important role in modulating immunity, anti-inflammation, cancer, angiogenesis, and tissue repair [8]. Numerous preclinical and medical studies have demonstrated the therapeutic possibilities of MSCs in ARDS [9–16]. However, MSCs MSC therapies also have risks, such as tumour induction, and their safety is questionable [17]. EVs are derived from cells, secreted by almost all types of cells, and play an important role in cellular communication [18–20]. EVs include lipids, proteins, and nucleic acids [18]. According to the different sources, sizes, and surface marks of EVs, they can be divided into apoptotic bodies (ABs), microvesicles (MVs), and exosomes. ABS is generally considered vesicles containing intracellular contents during apoptosis (1000-5000 nm) [21]; while MVs are thought to be secretory vesicles from the plasma membrane by the outward budding (100-1000 nm) [22]. Exosomes are formed from the maturation of intraluminal vesicles as multivesicular bodies before their fusion with the plasma membrane for secretion (30-100 nm) [20–22]. However, due to the overlapping sizes and lack of specific surface markers in this classification, the International Society for Extracellular Vesicles (ISEV) introduced new guidelines in 2018 to provide a more precise categorization of EVs. According to the updated guidelines, EVs are now divided into small EVs (Small extracellular vesicles,sEVs) and large EVs (Large extracellular vesicles,LEVs). sEVs refer to small vesicles originating from intracellular compartments such as endosomes, multivesicular bodies, or the endoplasmic reticulum, typically ranging in diameter from 30 to 150 nm, and they can be isolated using techniques like ultracentrifugation. On the other hand, LEVs are large vesicles derived from cell membrane budding or extracellular matrix, with diameters generally exceeding 200 nm, and they can be separated through filtration methods [23]. This novel classification approach based on the different cellular origins of EVs enables a more accurate description of their distinct characteristics and functions.

We conducted a meta-analysis based on data collected from preclinical animal models of ALI to systematically assess the efficacy of EVs as a potential treatment.

### Materials and methods

We use the PRISMA statement to conduct this meta-analysis [24]. The study protocol was registered on the International Prospective Register of Systematic Reviews (PROSPERO): (CRD42022368159).

### Study selection

Two investigators (ZXF and CZY) searched and screened the applicable literature independently and then assessed the titles and abstracts of every retrieved article to decide which required further assessment. Disagreements between the investigators were resolved with a discussion or adjudicated by another reviewer (ZMH).

The inclusion criteria were as follows: (1) any animal models with ALI; (2) any studies intervened with various cell-derived EVs; (3) negative control (treatment without EVs). The primary outcome was the lung injury score. Secondary outcomes included inflammatory factors IL-1β, IL-6 and TNF-α, anti-inflammatory factor IL-10, W/D ratio of the lung, total protein in BALF, white blood cell counts in BALF, and neutrophil counts in BALF.

The exclusion criteria were as follows: (1) the animal models without ALI; (2) the data were repeated; (3) incomplete information; (4) review, letter, commentary, correspondence, case report, conference abstract, expert opinion, or editorial.

### Data extraction

The data were extracted with the aid of two unbiased reviewers (ZXF and CZY) in a standardized way. For discrepancies, a third reviewer (ZMH) extracted them again.

The following data were collected: first author, country or region, type of ALI model, species, treatment time, measurement time and EV cell origins, diameter, and dose. We extracted data from graphics based on Engauge Digitizer version 4.1 software [25, 26].

To extract data from the study, we saved all relevant screenshots from the results as images and uploaded these images to the program. The first step was to determine what type of graph we were analyzing. Secondly, three known values were assigned to three points on the axis to calibrate it. Then, we directly clicked on each point on the graph to get its exact coordinates and used those coordinates to calculate its mean and standard deviation.

### Quality assessment

Two independent authors (ZXF and ZMH) evaluated each study's methodological quality with a Collaborative Approach to Meta-Analysis and a Review of Animal Data from Experimental Studies (CAMARADES) 10-item checklist [27]. (Table 2).

### Statistical analysis

All statistical analyses were conducted using RevMan version 5.3 and State 15.1 statistical software. It was considered statistically significant when the P < 0.05 (two-tailed). Continuous outcomes were expressed as standardized mean differences (SMD) with a 95% confidence interval (95%CI). Using the I^2^ statistic, heterogeneity was assessed among studies. An I^2^ > 50% indicates significant heterogeneity [28]. In order to inspect conceivable between-study heterogeneity and to discover different viable confounding factors, subgroup, sensitivity, and meta-regression analyses were carried out. The publication bias was detected through funnel plots and Egger's test. If publication bias was indicated, we recalculated pooled risk estimates by including those missing studies from the Trimfill method.

## Results

### Search results and study characteristics

**Fig. 1:**
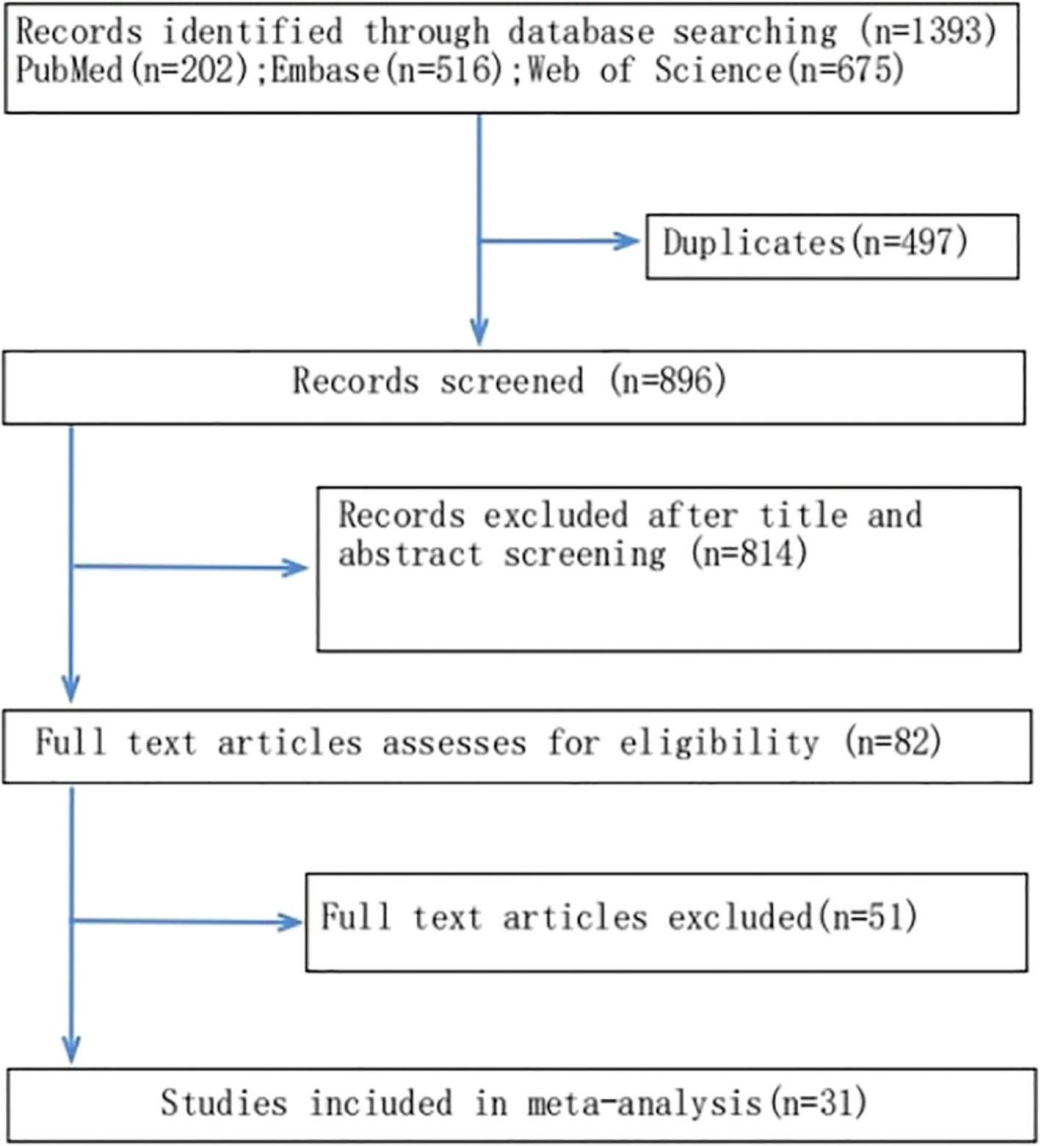
The diagram of the literature search process

Figure 1 is the diagram of the literature search process; 31 studies conform to the inclusion criteria [14, 29–57]. The main characteristics of these studies are presented in Table 1. All of these studies were published between 2010 and 2022. Among these studies, the number of bone marrow mesenchymal stromal cell (BMSC)-EVs is 16 [13, 14, 33, 36–38, 40–43, 45, 46, 49, 50, 53, 54]. 5 used adipose-derived mesenchymal stromal cell (ADMSC)-EVs [30, 34, 35, 44, 52]. 3 used human umbilical Wharton’s jelly mesenchymal stromal cell (WJ-MSC)-EVs [31, 32, 57]. 3 used alveolar macrophages (AMs)-EVs [47, 55, 56]. 1 used human umbilical cord mesenchymal stromal cell (UCMSC)-EVs [49]. 1 used human umbilical vein endothelial (UVLC)-EVs [29]. 1 used bone endothelial progenitor cells (EPC)-EVs [51]. 1 used Alveolar Epithelial Cell [39].

**Table 1:** Abbreviations: ADMSC adipose derived mesenchymal stromal cells, BMSC bone marrow mesenchymal stromal cells, C control group, CLP caecal ligation and puncture,EPC endothelial progenitor cell, EVs extracellular vesicles, MVs microvesicles, NR not reported, SD Sprague–Dawley, T treatment group, UCMSC umbilical cord mesenchymal stromal cells, UVEC umbilical vein endothelial cells, WJMSC Wharton’s jelly mesenchymal stromal cells BLM: bleomycin LPS: lipopolysaccharide SwIV: swine/MN/08; H1N1 MDR-P: multidrug-resistant PA

Author	Country or region	Injury type	Species	Sex	Cell source of EVs	Diameter (nm)	Administration methods	Therapy time	Measurement time	Dose	Main finding
Buesing, Keely L [29]	USA	it lps	C57BL/6 J	male	UVECl	< 300	iv	24 h	24 h	20,000/ml	EMPs are significant contributors to ALI via inflammatory cytokines, resultant neutrophil recruitment, and ultimately increases in MPO levels in the lung tissue
Chang, Chia-Lo [30]	China	CLP	SD rats	male	ADMSCs	30–90	iv	117 h	120 h	50ug	Not only localized but also systemically inflammatory reactions were elicited by SS. Apoptotic ADMSC-derived exosomes might be inferior to healthy ADMSC-derived exosomes for reducing the multi-organ damage and mortality rate in rodents after SS
Chen, W [31]	China	it BLM	SD rats	male	WJMSCs	200	it	24 h	48 h	NR	HGF mRNA partly mediated the therapeutic effects of MSC-MVs on ALI in mice induced by BLM via PI3K-Akt-mTOR activation
Chen, W [32]	China	it BLM	SPF rats	male	WJMSCs	200	it	5d	7d	10ul	WJMSC-MV-transferred miR-100 mediated, at least in part, the therapeutic effect of WJMSC-MVs in ALI through restoring mTOR signalmediated inhibition of autophagy
Deng, H [33]	China	injected intraperitoneally lps	C57BL/6 J	male	BMSCs	80–150	it	23 h	24 h	100ug	BMSCs-derived exosomes inhibited the inflammatory response and regulated macrophage polarization possibly through inhibiting HIF-1amediated glycolysis
Deng, H [34]	China	injected intraperitoneally lps	C57BL/6 J	male	ADMSCs	40–150	it	23 h	24 h	100ug	Exosomes derived from hMSCs from adipose tissue exhibited particularly strong effects in promoting macrophage M2 polarization, inhibiting proinflammatory cytokine production
Huang, R [35]	China	it lps	C57BL/6 J	NR	ADMSCs	50–400	iv	47.5 h	48 h	100ug	Aging MSC-EVs had higher levels of miR-127-3p and miR-125b-5p (M1) compared with young MSC-EVs. This finding might explain the observed difference in M2 macrophage polarization between aging and young MSC-EVs
Kaspi, Haggai [36]	USA	it lps	C57BL/6 J	female	BMSCs	146	it	69 h	72 h	50ul	Exo MSC-NTF reduced neutrophil count, TF, and fibrin, in the lung tissue, thereby interrupting a disease cascade that may explain the early lung recovery or the prevention of damage following intratracheal exosome treatment.
Khatri, M [37]	USA	it SwIV	White-Duroc crossbred pigs	NR	BMSCs	100	it	60 h	72 h	80ug/Kg	EVs derived from porcine BM-MSCs inhibit the HA activity of influenza viruses and SwIV replication and virus-induced apoptosis in LECs
Li, Qing-Chun [38]	China	struck the chest	SPF rats	NR	BMSCs	30–50	iv	7d30min	7d	25ug	MiR-124-3p transferred by MSC-derived exosomes inhibits the expression of P2X7, thus alleviating OS injury and inflammatory response in rats with traumatic ALI
Liu, F [39]	China	CLP	SD rats	NR	Alveolar Epithelial Cel	100	it	24 h	24 h	2 mg/Kg	Exosome-shuttled miR-92a-3p mediated the crosstalk between AECs and AMs, which contributes to macrophage activation by inhibiting PTEN expression and regulating the activation of the NF-kB signalling pathway
Liu, Jian-Hua [40]	China	it lps	C57BL/6 J	male	BMSCs	50–200	iv	44 h	48 h	100ug	Exosomal miR-132-3p ameliorated LPSinduced ALI via targeting TRAF6 and inactivating PI3K/Akt signalling
Liu, X [41]	China	it lps	SD rats	male	BMSCs	63–269	it	20 h	24 h	50ul	BMSC-derived exosomes alleviate LPSinduced autophagy stress of alveolar macrophages, at least partly, via delivering exosomal miR-384-5p to alveolar macrophages
Mao, Guan-chao [42]	China	inject the Sulfur mustard on the surfaces	ICR mice	male	BMSCs	30–100	iv	96 h	120 h	20 mg/Kg	The antiapoptotic and barrier-regenerating effects of BMSC-Exs may be mediated by the upregulation of GPRC5A expression in recipient cells, which activates the YAP pathway, leading to the promotion of Bcl-2 and junction protein expression and relocalization
Monsel, Antoine [14]	France	it lps	C57BL/6 J	male	BMSCs	200	it	20 h	24 h	60ul	MV released from BMSCs improved survival from E.coli pneumonia in mice. This was associated with enhanced phagocytosis of bacteria by human monocytes with a reduction in inflammation and increased ATP levels in alveolar epithelial type 2 cells
Morrison, T. J [43]	UK	intranasally lps	C57BL/6 J	male	BMSCs	< 4000	intranasally	20 h	24 h	NR	MSCs modulate human macrophages towards decreased production of proinflammatory cytokines, increased expression of the M2 phenotype marker CD206 and enhanced phagocytic capacity. MSC-EVs carrying mitochondria are responsible for these effects through the promotion of oxidative phosphorylation in macrophages
Shi, Meng-meng [44]	China	it MDR-P	C57BL/6 J	male	ADMSCs	50–400	it	20 h	24 h	NR	MSCs and miR-466 promoted macrophage polarization toward Type 2 phenotype through TIRAPMyD88-NFκB axis
Silva, J. D [45]	Brazil	it lps	C57BL/6 J	female	BMSCs	193.7–670.1	iv	24 h	48 h	50ul	MSCs yielded greater overall improvement in ARDS in comparison to EVs derived from the same number of cells and regardless of the preconditioning status
Silva, J. D [46]	UK	it lps	C57BL/6 J	male	BMSCs	100–700	iv	20 h	24 h	5*10^5 particles	MSC-EVs downregulate LPS-induced inflammatory response and attenuate mitochondrial dysfunction in human PCLSs. Therapeutic effect of MSC-EVs on the restoration of barrier integrity is mediated by mitochondrial transfer
Soni, S [47]	UK	it lps	C57BL/6 J	male	Alveolar macrophage	< 1000	it	4 h	4 h	NR	MVs released in vitro from LPS-primed alveolar macrophages caused similar increases in MLE-12 ICAM-1 expression, which was mediated by TNF
Tang, Xiao-Dan [48]	China	it lps	C57BL/6 J	male	BMSCs	200	it	48 h	48 h	30ul	The therapeutic effects of microvesicles in acute lung injury, and their immunomodulatory properties on macrophages were partly mediated through their content of Angiopoietin-1 mRNA
Varkouhi, Amir K [49]	Canada	it lps	SD rats	male	UCMSCs	47.7 ± 25.2	it	48 h	48 h	NR	The mechanistic insights into the actions of mesenchymal stromal cell–derived extracellular vesicles, namely enhancement of macrophage phagocytosis and killing of bacteria and restoration of endothelial nitric oxide synthase, which may restore capillary endothelial barrier function
Wang, Jiangmei [50]	China	it lps	C57BL/6 J	NR	BMSCs	50–150	it	48 h	48.5 h	50ug	Mesenchymal stem cell–derived extracellular vesicles mitigate acute lung injury at least partially via transferring miR-27a-3p to alveolar macrophages. miR-27a-3p acts to target NFKB1 and is a crucial regulator of M2 macrophage polarization
Wu, X [51]	China	it lps	SD rats	male	Bone endothelial progenitor cells	30–110	iv	48 h	48 h	100ug	MiR-126 of exosomes probably modulated the proliferation, migration and tube formation of ECs partly through directly inhibiting SPRED-1, so that to activate the RAF/ERK signaling
Xia, L [52]	China	it lps	C57BL/6 J	female	ADMSCs	50–150	iv	20 h	24 h	10ug	AdMSC-Exos can effectively donate mitochondria component improved macrophages mitochondrial integrity and oxidative phosphorylation level, leading to the resumption of metabolic and immune homeostasis of airway macrophages and mitigating lung inflammatory pathology
Xu, J [53]	China	it lps	C57BL/6 J	male	BMSCs	30–100	iv	48 h	48 h	100ug	Exosomes and miR-150 reduced inflammation and lung edema while maintaining the integrity of the alveolar structure. They also mitigated microvascular endothelial cell injury by regulating the caspase-3, Bax/Bcl-2, and MAPK signaling
Xu, N [54]	China	expose to the phosgene	SD rats	male	BMSCs	50–200	iv	24 h	24 h	50ul	MSC derived exosomes exerted the therapeutic effects on phosgene-induced ALI through inhibiting MMP-9 synthesis and up-regulating SP-C
Xu, Xinyi [55]	China	it lps	C57BL/6 J	male	Alveolar macrophage	200	it	24 h	24 h	NR	Secretory Autophagosomes from Alveolar Macrophages Exacerbate Acute Respiratory Distress Syndrome by Releasing IL-1β
Zhang, L [56]	China	it lps	C57BL/6J	NR	Alveolar macrophage	100	it	4 h	4 h	50ug	According to our results, inflammatory AM-derived MVs may potentially contribute to lung injury and pulmonary edema, thereby indicating a potential novel therapeutic approach against ALI/ARDS based on AM-MVs
Zhao, R [57]	China	it lps	C57BL/6 J	male	WJMSCs	82.5–164.1	iv	20 h	24 h	50ug	The inhalation of MSC-EVs presented better performance than those administered via tail vein injection for the treatment of ALI, as well as exhibited robust antiinflammatory and antioxidative activity in LPS-stimulated cells and animal models
Zhu, Ying-gang [14]	China	it lps	C57BL/6 J	male	BMSCs	200	it	36 h	48 h	30ul	Human MSC-derived MVs were therapeutically effective following E. coli endotoxin-induced ALI in mice in part through the expression of KGF mRNA in the injured alveolus

The main animal models were mice and rats; in one study the animal model was a pig. ALI induced by intratracheal injection of lipopolysaccharide or E.coli was the most common method in these studies. At the same time, there are other research methods, such as intratracheal injection of Bleomycin, H1N1 virus, pseudomonas Aeruginosa, cecal ligation and perforation, and trauma. Different studies had different administration methods, intervention times, and intervention doses. In addition, there was a significant difference in the time point at which the results were assessed, with most studies completed within two days and a few lasted one week. All the included studies were peer-reviewed publications. All the experimental animals were randomly assigned to the intervention group and the control. The quality assessment of all studies is shown in Table 2.

**Table 2:** Abbreviation: 1,peer-reviewed journal; 2,temperature control; 3,animals were randomly allocated; 4,blind established model; 5, blinded outcome assessment; 6,use of anesthetic without significant intrinsic vascular protection activity; 7, appropriate animal model (diabetic, advanced age or hypertensive); 8,calculation of sample size; 9,statement of compliance with animal welfare regulations; 10,statement of potential conflict of interests

Study	publication year	1	2	3	4	5	6	7	8	9	10	Total
Buesing, Keely L [29]	2011	1	0	1	0	0	1	1	0	1	0	5
Chang, Chia-Lo [30]	2018	1	1	1	0	1	1	1	0	1	0	7
Chen, W. X [31]	2019	1	1	1	0	0	1	1	0	1	1	7
Chen, W. X [32]	2020	1	1	1	0	0	1	1	0	1	1	7
Deng, H. [33]	2020	1	1	1	0	0	1	1	0	1	0	6
Deng, H [34]	2022	1	1	1	0	0	1	1	0	1	1	7
Huang, R. [35]	2019	1	1	1	0	0	1	1	0	1	1	7
Kaspi, Haggai [36]	2021	1	1	1	0	0	0	1	0	1	0	5
Khatri, M. [37]	2018	1	1	1	0	0	1	1	0	1	1	7
Li, Qing-Chun [38]	2019	1	1	1	0	0	1	1	0	1	1	7
Liu, F. [39]	2021	1	1	1	0	0	1	1	0	1	0	6
Liu, Jian-Hua [40]	2021	1	1	1	0	0	1	1	0	1	1	7
Liu, X. [41]	2021	1	1	1	0	0	1	1	0	1	1	7
Mao, Guan-chao [42]	2021	1	1	1	0	0	0	1	0	1	1	6
Monsel, Antoine [14]	2015	1	1	1	0	0	0	1	0	1	1	6
Morrison, T. J. [43]	2017	1	1	1	0	0	1	1	0	1	1	7
Shi, Meng-meng [44]	2021	1	1	1	0	0	1	1	0	1	1	7
Silva, J. D. [45]	2019	1	1	1	1	1	1	1	0	1	1	9
Silva, J. D. [46]	2021	1	1	1	0	0	1	1	0	1	1	7
Soni, S. [47]	2016	1	1	1	0	1	1	1	0	1	1	8
Tang, Xiao-Dan [48]	2017	1	1	1	0	0	1	1	0	1	1	7
Varkouhi, Amir K. [49]	2019	1	1	1	0	0	1	1	0	1	1	7
Wang, Jiangmei [50]	2020	1	1	1	0	0	1	1	0	1	1	7
Wu, X. [51]	2018	1	1	1	0	0	1	1	0	1	1	7
Xia, L. [52]	2022	1	1	1	0	0	1	1	0	1	1	7
Xu, J. [53]	2022	1	1	1	0	0	1	1	0	1	1	7
Xu, N. [54]	2019	1	1	1	0	0	0	1	0	1	1	6
Xu, Xinyi [55]	2022	1	1	1	0	0	0	1	0	1	1	6
Zhang, L. [56]	2022	1	1	1	1	1	1	1	0	1	1	9
Zhao, R. [57]	2022	1	1	1	0	0	1	1	0	1	1	7
Zhu, Ying-gang [14]	2014	1	1	1	0	0	1	1	0	1	1	7

### Meta-analysis: ALI with EVs group versus ALI without EVs group (control group)

#### Heterogeneity text

**Fig. 2:**
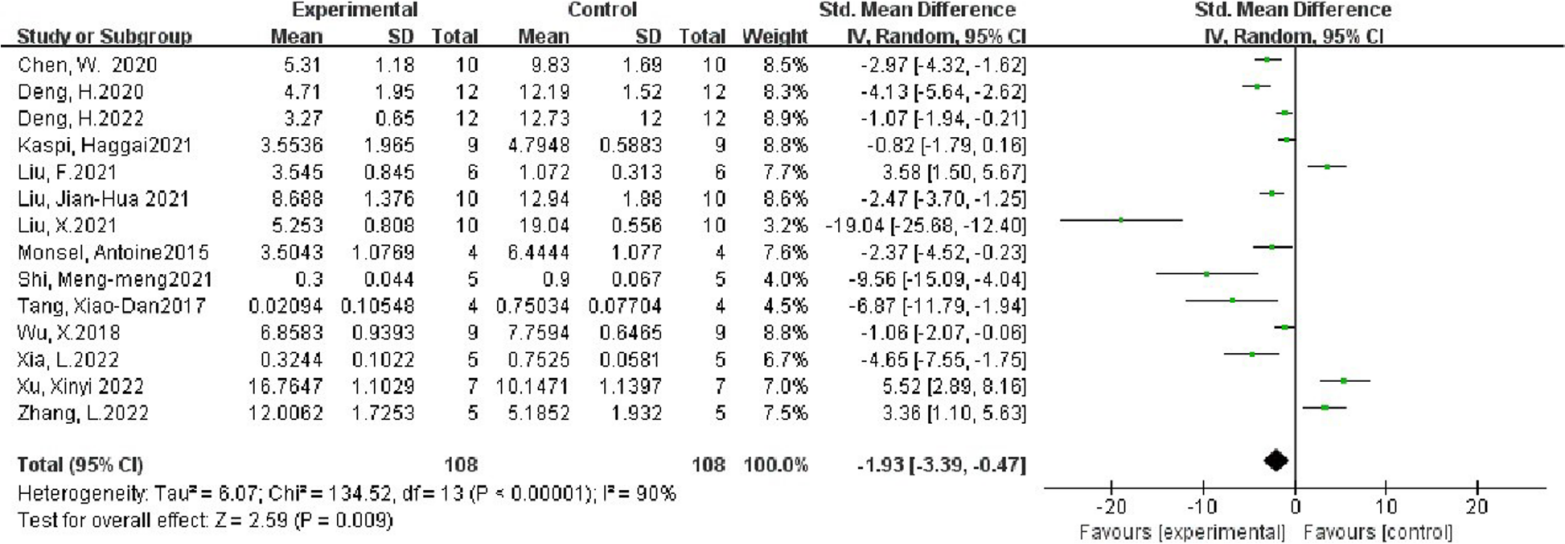
Main outcome of the meta-analyses of the ALI with EVs group compared with the ALI without EVs control group. Main outcome is lung injury score. The size of each square represents the proportion of information given by each trial. Crossing with the vertical line suggests no diference between the two groups. *ALI* acute lung injury, *IV* inverse variance, *CI* confdence interval, *df* degree of freedom

There were 14 articles in this study. After the Heterogeneity test, I^2^ = 90% > 50%, and Q test *P* < 0.1, the articles were considered to have a strong heterogeneity. The reasons for heterogeneity should be investigated. Based on the data of this study, a subgroup analysis was carried out from the aspects of the origin of extracellular vesicles, the species of experimental animals, intervention time, administration methods, and different scoring system of lung injury score. For the overall 14 articles, random effects were selected for meta-analysis, and the results were as follows (Fig. 2).

The results of the meta-analysis showed that the lung injury score of the experimental group was 1.93 lower than that of the control group, and the degree was statistically significant (*P* < 0.05).

#### Sensitivity analysis

**Fig. 3:**
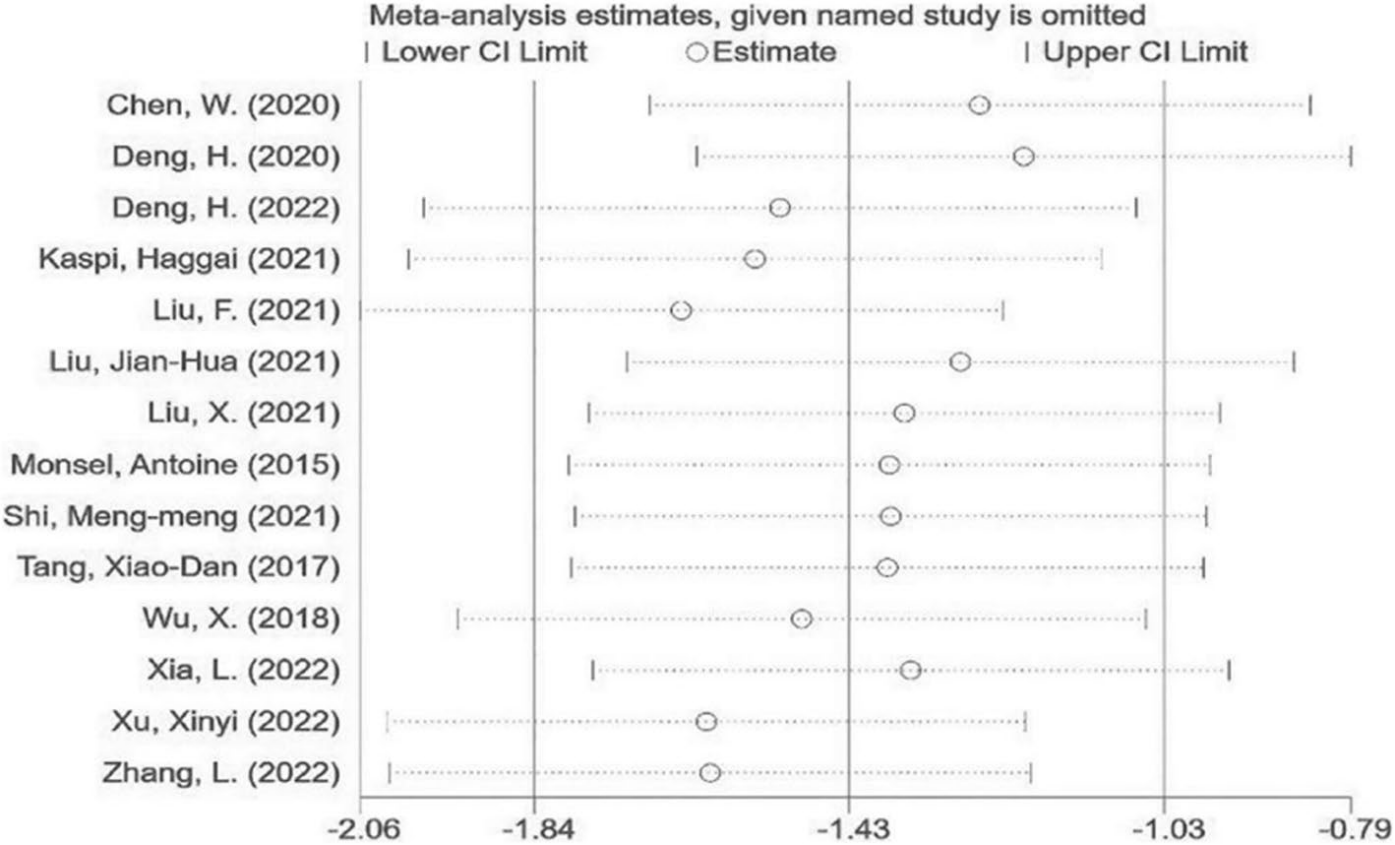
Sensitivity analysis of Lung Injury Score 14 studies were included in the sensitivity analysis, and after excluding each study, the combined effects of the remaining studies were within 95% confidence interval of the total effect

A sensitivity analysis of 14 articles was carried out, and the results were as follows (Fig. 3). As can be clearly seen from the above figure, none of the studies significantly caused heterogeneity, and the stability of the overall study was high. Further subgroup analysis was carried out.

#### Subgroup analysis

**Fig. 4:**
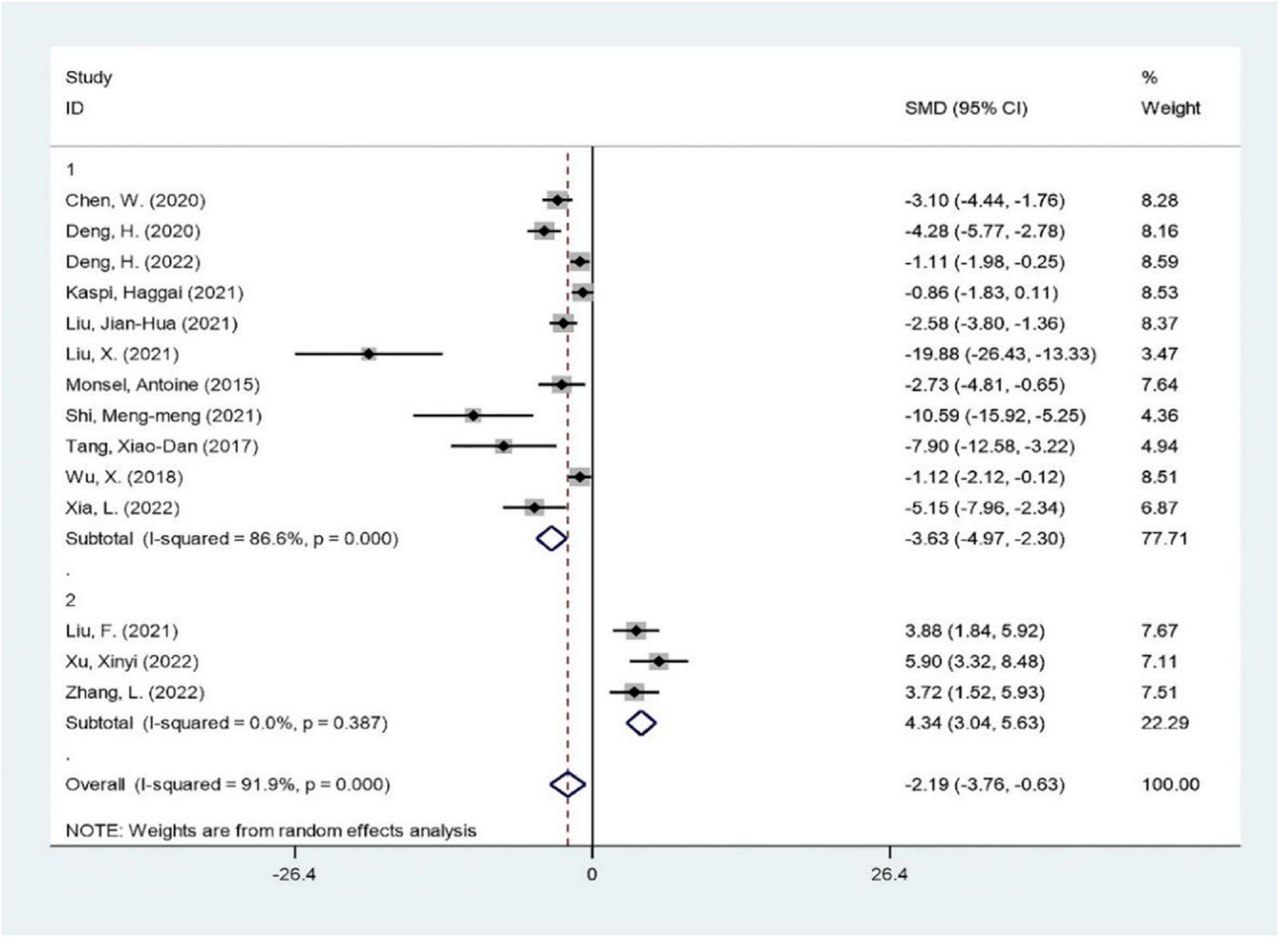
subgroup analysis The subgroup meta-analysis of lung injury score that compares different origins of extracellular vesicles. The analysis didn’t detect any statistically significant difference among the stem-progenitor cell extracellular vesicles(1), non-stem progenitor cell extracellular vesicles(2)

The 14 articles were divided into two groups according to the source of extracellular vesicles. The results of the meta-analysis were as follows (Fig. 4). Based on the subgroup analysis above, the heterogeneity between the two groups was extreme, reaching a high degree of heterogeneity. Among them, the efficacy of stem-progenitor cell extracellular vesicles was -3.63, which was significant (Z = 5.34, *P* < 0.05). This means that stem progenitor cell extracellular vesicles significantly reduce the severity of acute lung injury to a large extent. Secondly, there was no heterogeneity in the group of non-stem progenitor cell extracellular vesicles, and the efficacy amount was 4.34 (Z = 6.55, *P* < 0.05). These results suggest that the extracellular vesicles of non-stem progenitor cells significantly increase the severity of acute lung injury. However, that the different sources of extracellular vesicles are the cause of heterogeneity cannot be explained.

**Fig. 5:**
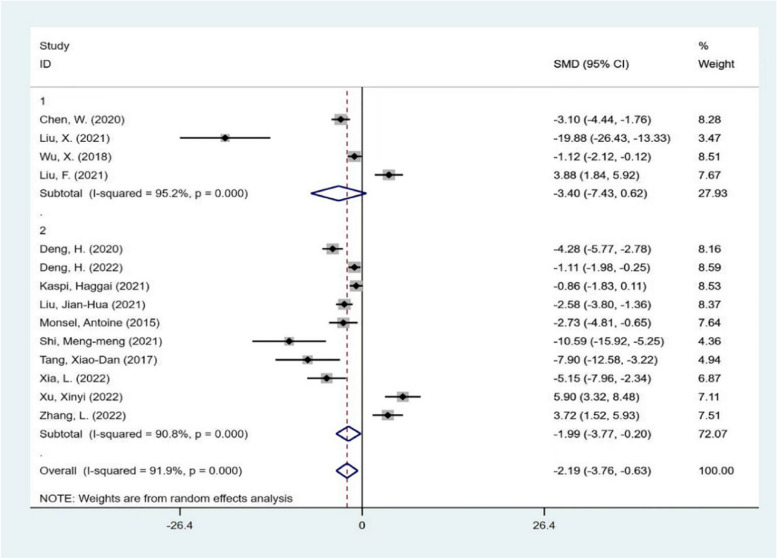
subgroup analysis The subgroup meta-analysis of lung injury score that compares different species of animals. The analysis didn’t detect any statistically significant difference among the SD rats(1), C57BL/6J mice(2)

**Fig. 6:**
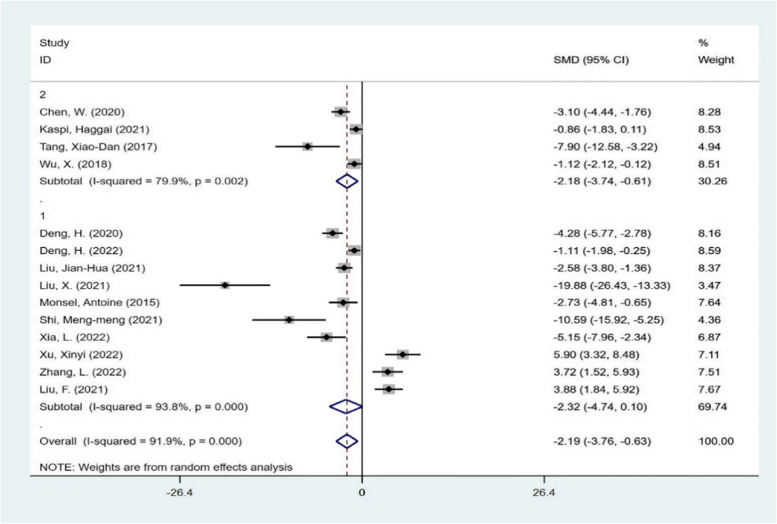
subgroup analysis The subgroup meta-analysis of lung injury score that compares different intervention time. The analysis didn’t detect any statistically significant difference among the intervention time less than 48 h (1), intervention time more than 48 h (2)

**Fig. 7:**
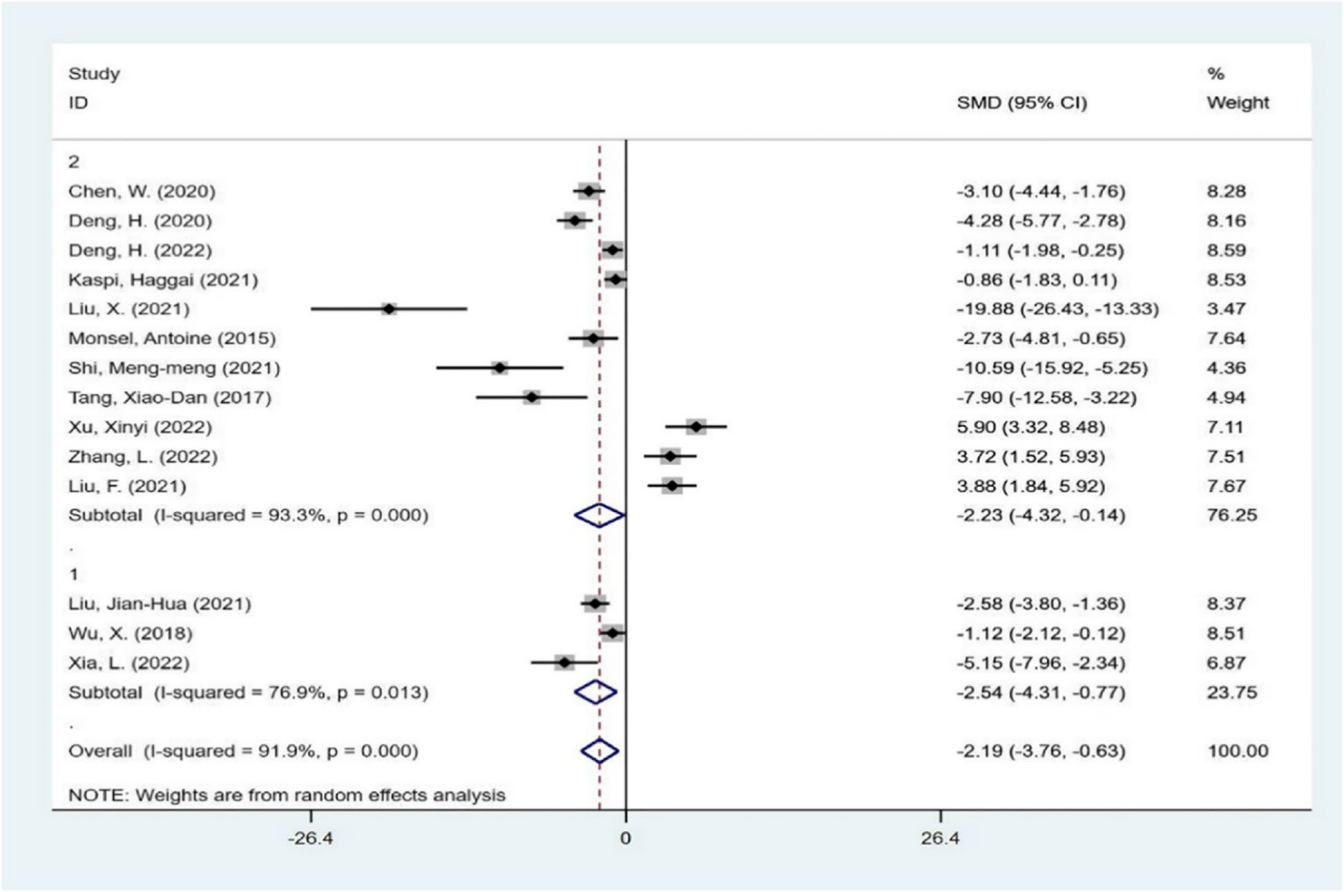
subgroup analysis The subgroup meta-analysis of lung injury score that compares different administrations. The analysis didn’t detect any statistically significant difference among the intravenous administration (1), intratracheal administration(2)

**Fig. 8:**
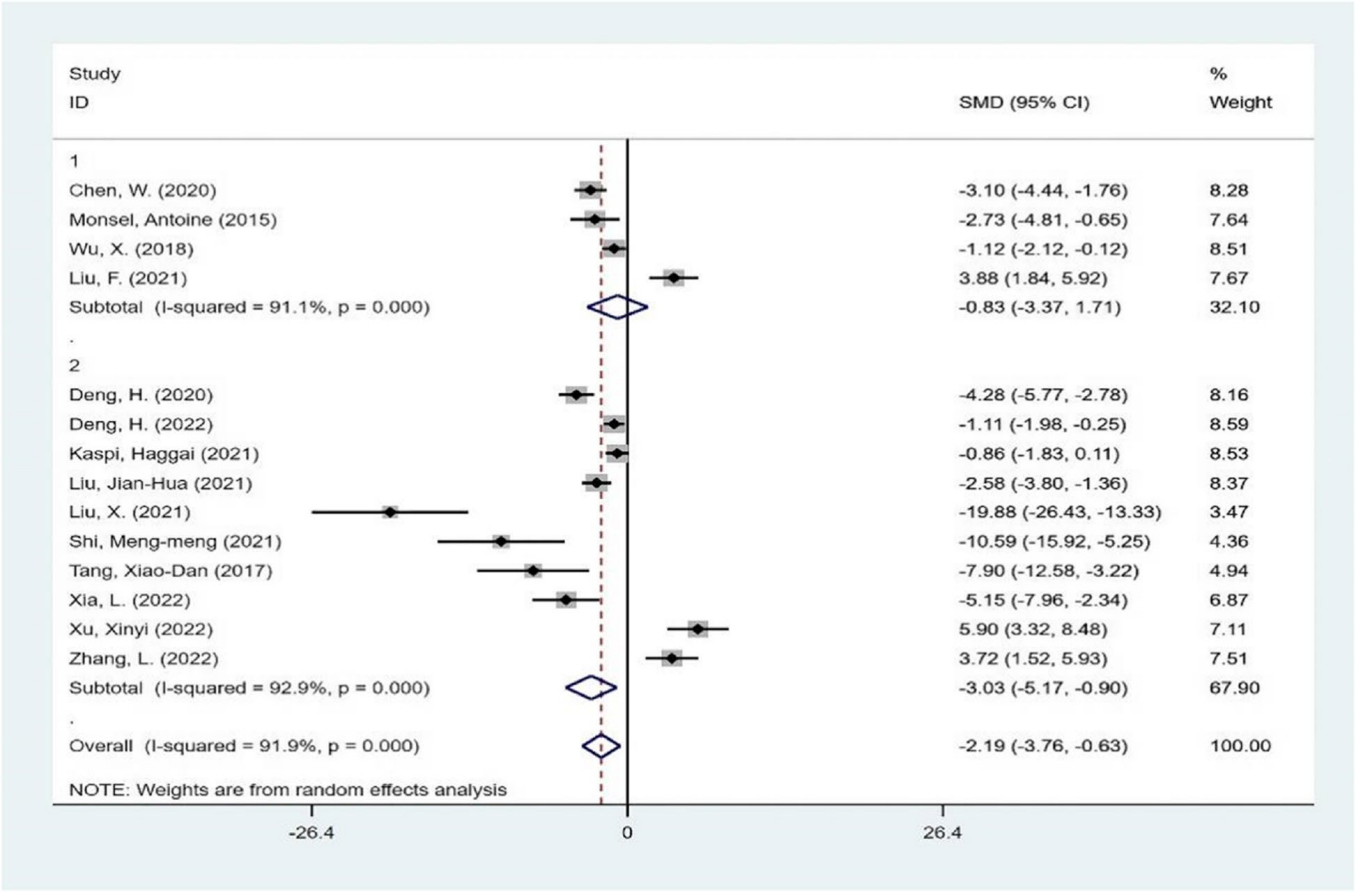
subgroup analysis The subgroup meta-analysis of lung injury score that compares different scoring system. The analysis didn’t detect any statistically significant difference among the semi-quantitative grading system(1), the lung injury scoring system(2)

Then, the subgroup analysis was carried out from the aspects of experimental animal species, intervention time, intervention mode and different scoring system of lung injury score. The results are shown in Figs. 5, 6, 7, and 8. Each subgroup shows a high degree of heterogeneity, and the combined results also show high heterogeneity. The results of subgroup analysis do not support that the type of experimental animals, intervention time, intervention mode and different scoring system of lung injury score are the causes of heterogeneity. Next, meta-regression was used to investigate the source of heterogeneity.

### Multivariable meta-regression analysis

**Fig. 9:**
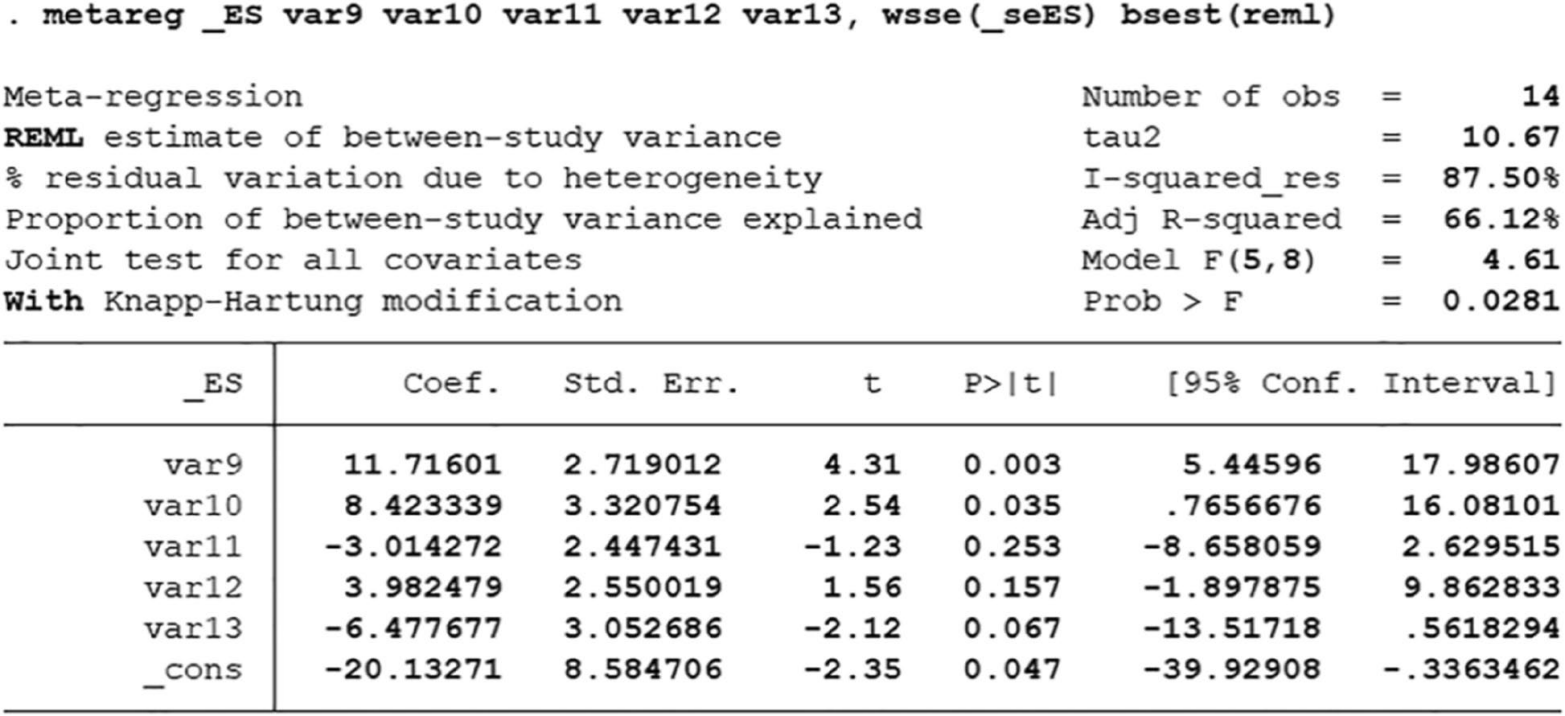
multivariable meta-regression analysis P < 0.05 Considering this variable is the cause of heterogeneity.(var9: extracellular vesicles are primarily derived from stem progenitor cells and non-stem progenitor cells; var10: experimental animals are mainly divided into mice and rats; var11: administration is divided into intratracheal administration and intravenous administration; var12: intervention time is divided into whether the intervention time is more than 48 h or not; var13: different scoring system of the lung injury score is divided into the semi-quantitative grading system and the lung injury scoring system)

From the above table (Fig. 9), we can see that the independent variable "different sources of extracellular vesicles "and" the species of experimental animals" can significantly affect the efficacy (*P* < 0.05), while the other independent variables—the mode of administration, the time of intervention and the different scoring system of the lung injury score—have no statistical effect. Overall, we believed that the different sources of extracellular vesicles and the species of experimental animals are the cause of greater heterogeneity. Var9 extracellular vesicles are primarily derived from stem and non-stem progenitor cells; var10 experimental animals are mainly divided into mice and rats; var11 administration is divided into intratracheal administration and intravenous administration; var12 intervention time is divided into whether the intervention time is more than 48 h or not; var13 different scoring system of the lung injury score is divided into the semi-quantitative grading system [58] and the lung injury scoring system [59].

### Publication bias

**Fig. 10:**
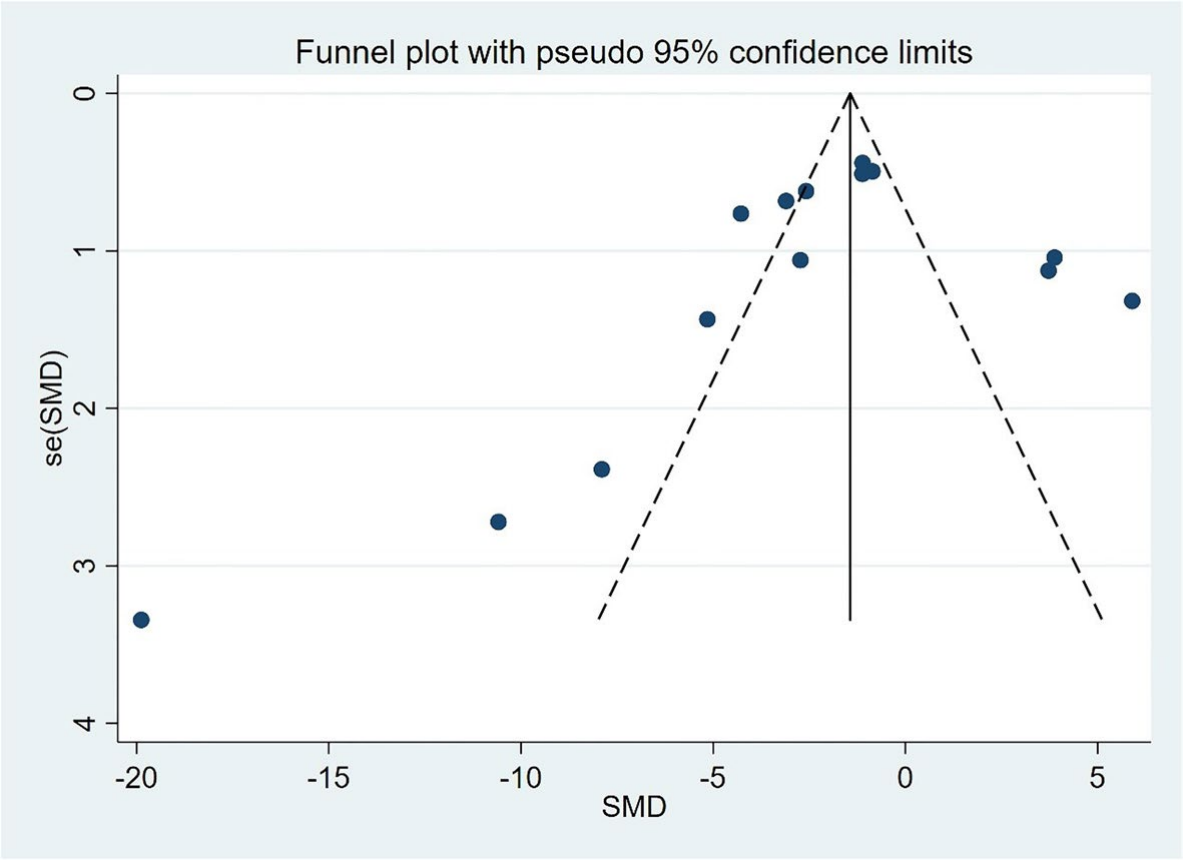
Funnel plots

The results were as follows (Fig. 10). From the figure, it can be seen clearly that the funnel diagram of this study is basically symmetrical, and the Egger bias test and the Begg bias test were carried out at the same time. The Egger test showed that there was no significant publication bias (*P* = 0.390 > 0.05). The Begg test results are consistent with the Egger test results (*P* = 0.08 > 0.05). Therefore, it can be said that there is no publication bias in the literature of this study.

#### Primary outcome

Lung injury score is considered the primary outcome. Secondary outcomes included IL-1β, IL-6, TNF-α, IL-10, W/D ratio, total protein in BALF, BALF total cell counts, white blood cell counts, and BALF neutrophil counts.

The results showed that compared with the negative control group, stem progenitor cell extracellular vesicle therapy could significantly reduce the lung injury score. The standardized mean difference (SMD) = -3.63 95%CI [- 4.97, 2.30], *p* < 0.05 I^2^ = 86.6%, while the non-stem progenitor extracellular vesicle (alveolar epithelial cells and alveolar macrophages) remedy considerably increased the lung injury score. (SMD) = 4.34 95%CI [3.04, 5.63], *p* < 0.05 I^2^ = 0%. (Fig. 4).

#### Secondary outcomes

**Fig. 11:**
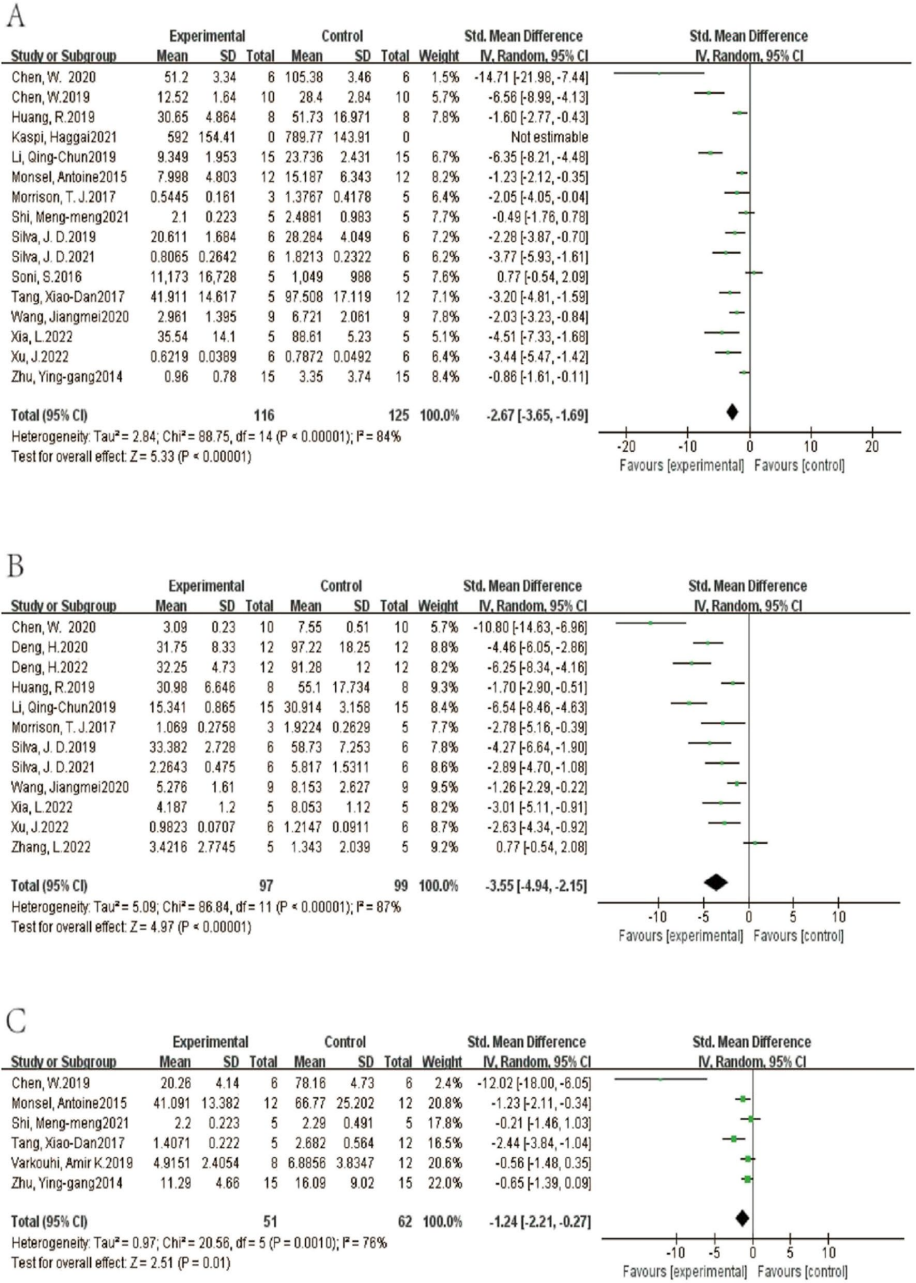
Secondary outcomes of the meta-analyses of the ALI with EVs group compared with the ALI without EVs control group. Secondary outcomes are BALF neutrophil count(A), total number of alveolar cells(B), alveolar leukocytes count(C). The size of each square represents the proportion of information given by each trial. Crossing with the vertical line suggests no diference between the two groups. ALI acute lung injury, IV inverse variance, CI confdence interval, df degree of freedom

A total of 16 studies reported BALF neutrophil counts. Compared with the control group, the number of neutrophils in the alveoli of the experimental group decreased (SMD = -2.67, 95% CI[-3.65,-1.69], *p* < 0.00001), I^2^ = 84% (Fig. 11A). There were 12 BALF cell count studies total. The results showed that compared with the control group, the total number of alveolar cells in the experimental group decreased (SMD = -3.55, 95% CI[-4.94,-2.15], *p* < 0.05, I^2^ = 87%) (Fig. 11B). The comprehensive results of six studies showed that extracellular vesicles could reduce the number of alveolar leukocytes compared with the control group (SMD = -1.24, 95% CI[-2.21,-0.27], *p* < 0.05) I^2^ = 76% (Fig. 11C).

**Fig. 12:**
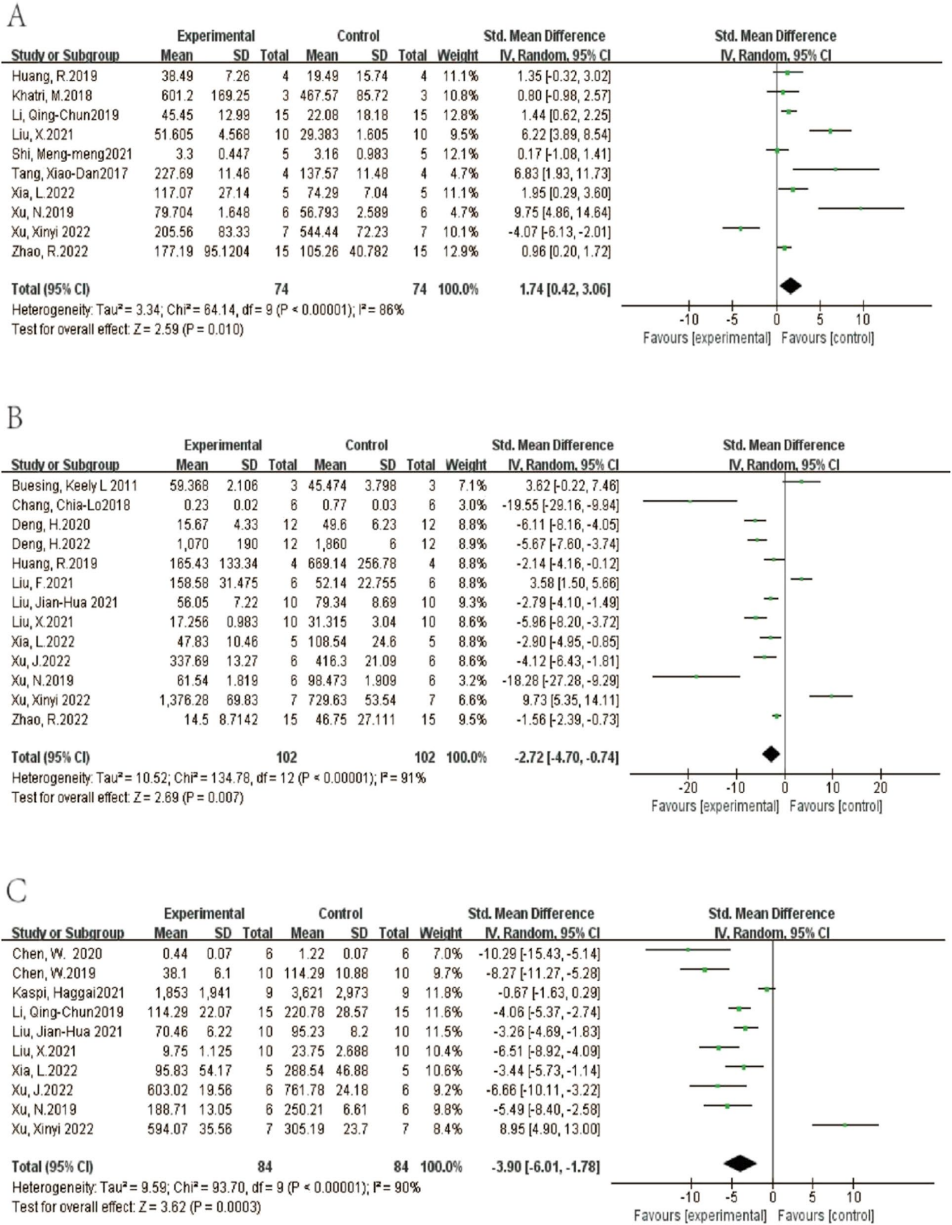
Secondary outcomes of the meta-analyses of the ALI with EVs group compared with the ALI without EVs control group. Secondary outcomes are IL-10(A), IL-1β(B), IL-6(C).The size of each square represents the proportion of information given by each trial. Crossing with the vertical line suggests no diference between the two groups. ALI acute lung injury, IV inverse variance, CI confdence interval, df degree of freedom

**Fig. 13:**
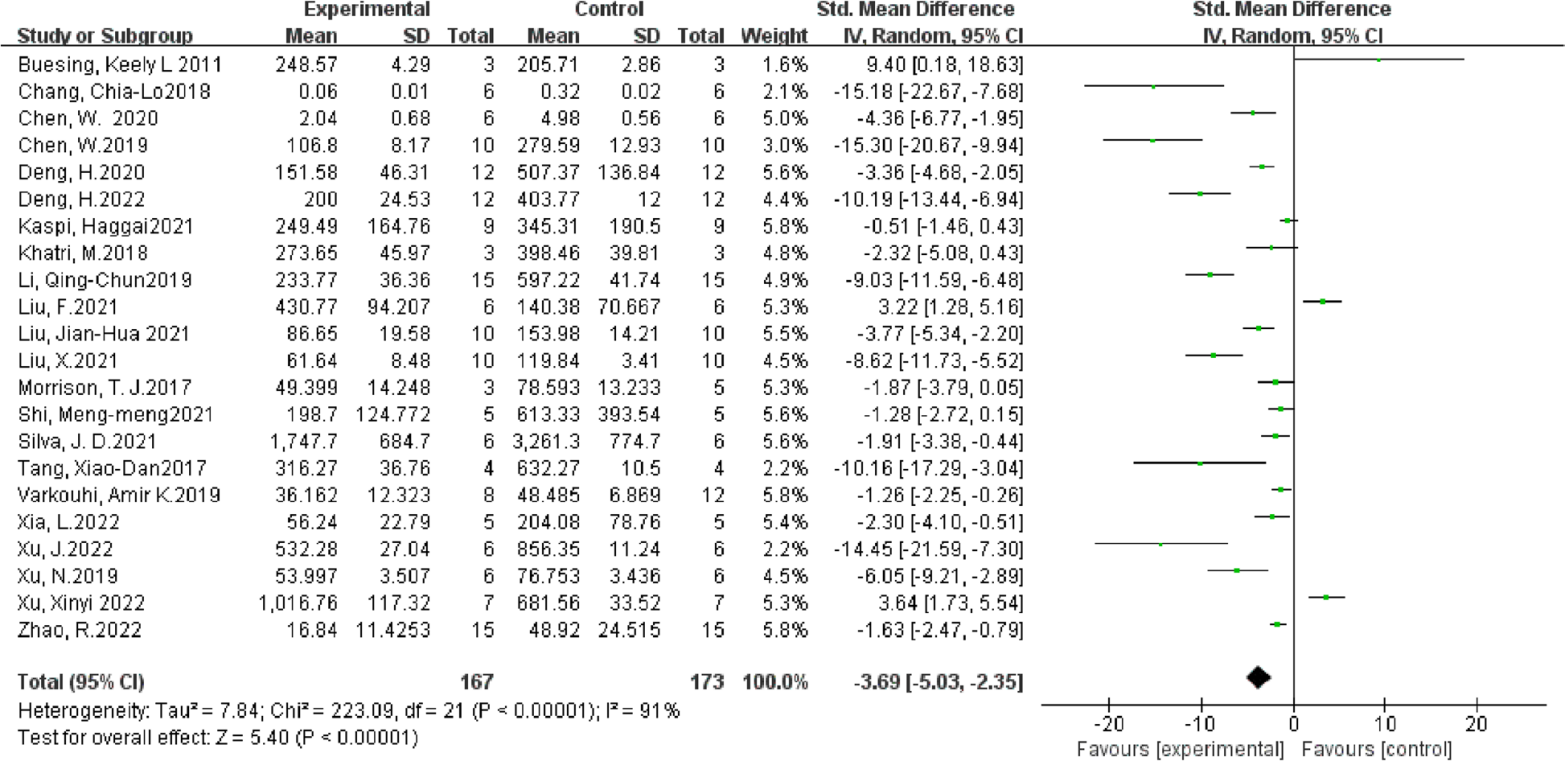
Secondary outcomes of the meta-analyses of the ALI with EVs group compared with the ALI without EVs control group. Secondary outcomes is TNF-a. The size of each square represents the proportion of information given by each trial. Crossing with the vertical line suggests no diference between the two groups. IV inverse variance, CI confdence interval, df degree of freedom

**Fig. 14:**
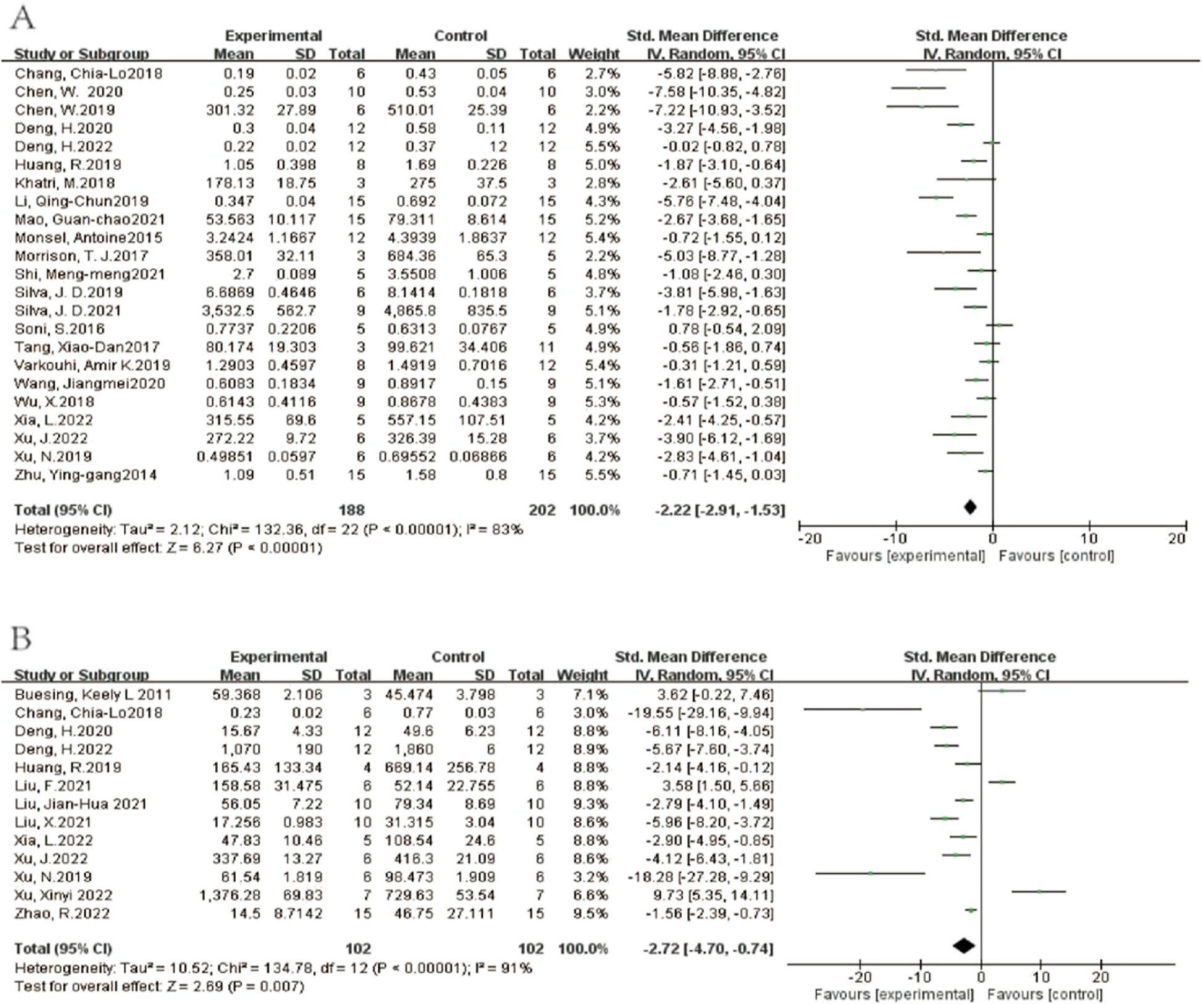
Secondary outcomes of the meta-analyses of the ALI with EVs group compared with the ALI without EVs control group. Secondary outcomes are total protein in BALF(A), W/D ratio(B).The size of each square represents the proportion of information given by each trial. Crossing with the vertical line suggests no diference between the two groups. ALI acute lung injury, IV inverse variance, CI confdence interval, df degree of freedom

A total of 10 studies investigated IL-10 in lung tissue. The results showed that compared with the ALI control group, EVs treatment could increase the level of IL-10. (SMD = 1.74, 95% CI [0.42, 3.06], *p* < 0.05, I^2^ = 86%) (Fig. 12A) A total of 13 studies reported IL-1β where their aggregate results showed that EVs could reduce the level of IL-1β compared with the control group. (SMD = -2.72, 95% CI [-4.70,-0.74], *p* < 0.05, I^2^ = 91%) (Fig. 12B) The comprehensive results of 10 studies show that EV can reduce the level of IL-6. (SMD = -3.90, 95% CI [-6.01,-1.78], *p* < 0.05, I^2^ = 90%) (Fig. 12C) In addition, 22 studies provided data on TNF-a, and the combined results show that EV can reduce the level of TNF-a (SMD = -3.69, 95% CI [-5.203,-2.35], *p* < 0.05, I^2^ = 91%) (Fig. 13). 23 studies investigated the level of total protein in BALF. The results showed that compared with the control group, EV ameliorated protein exudation. (SMD = -2.22, 95% CI [-2.91,-1.53], *p* < 0.05, I^2^ = 83%) (Fig. 14A) Compared with the control group, EV treatment can reduce W/D ratio (SMD = -2.74, 95% CI [-4.15,-1.30], *p* < 0.00001, I^2^ = 83%) (Fig. 14B).

## Discussion

This meta-analysis of 31 studies provides a comprehensive summary of the impact of EVs on preclinical animal models of ALI. Analysis of the combined data suggests that the attenuation of the inflammatory response and the improvement in lung function depend on the type of EVs involved.

Previous meta-analyses have shown that MSC-derived EVs reduce the inflammatory response of inflammatory cells, decrease lung permeability, decrease the activity of inflammatory mediators, and increase the activity of anti-inflammatory mediators in an animal model of ARDS, thereby attenuating lung injury and improving survival in an animal model of ARDS [60]. However, previous studies have not considered the effects of other sources of EVs on ALI/ARDS. In our meta-analysis, we incorporated extracellular vesicles (EVs) from diverse cellular origins to investigate their impact on lung injury scores, cell, and inflammatory factors in bronchoalveolar lavage fluid (BALF). Our findings offer valuable insights for future studies. Multiple regression analyses revealed that the cell source independently influenced the efficacy of EVs. Additionally, the choice of experimental animal introduced notable heterogeneity, while no statistically significant differences were observed among modes of administration, duration of intervention, and lung injury scoring systems. This meta-analysis demonstrates the mitigating effects of stem progenitor cell-derived extracellular vesicles (EVs) on ALI/ARDS severity in an animal model. The Lung Injury Score (LIS), a pivotal pathophysiological metric in clinical trials for assessing lung injury severity, was significantly reduced by stem progenitor cell-derived EVs, indicative of a diminished overall severity of lung injury. In addition, our study found that stem cell-derived EVs down-regulated the levels of inflammatory factors such as IL-1 β, IL-6, and TNF-a, and up-regulated the levels of IL-10, a traditional anti-inflammatory cytokine. Modulation of the balance of pro-inflammatory and anti-inflammatory cytokines by stem cell-derived EVs may be important for ameliorating lung injury and improving survival. In contrast, EVs from alveolar epithelial cells and macrophages exacerbate the extent of lung injury. The lung W/D ratio is a widely used index to assess pulmonary vascular permeability in animal experiments. In the present meta-analysis, the lung W/D ratio decreased, suggesting that stem cell-derived EVs increase lung water clearance. Also, our meta-analysis showed that neutrophils, leukocytes, and total protein in bronchoalveolar lavage fluid were reduced after intervention with stem cell EVs. This suggests that stem cell-derived EV therapy reduces the effects of lung tissue infection, vascular permeability and lung tissue damage. Numerous research have proven that RNAs carried by means of EVs are integral for their therapeutic characteristics [61, 62], and the proteins contained in EVs are also additionally associated with various biological functions in the human body. EVs are membrane-bound vesicles launched by way of all cell types, which are necessary data carriers for controlling angiogenesis, extracellular matrix remodelling, gene expression, inflammation, cell proliferation, cell migration, and morphogenetic elements [63–66].

As surface molecules, EVs can target receptors, facilitating signal transduction via receptor-ligand interactions. They undergo internalization through endocytosis, phagocytosis, and fusion with target cell membranes. With a composition akin to normal cell membrane and surface proteins, EVs are readily taken up by target cells, delivering their contents to the cytoplasm and thereby modulating the physiological state of recipient cells. This characteristic raises optimism regarding their potential as the next generation of drug transporters [67–70]. EV derived from stem cells reduces immunogenicity compared to stem cells and also reduces the dangers associated with cellular therapies such as cytokine release syndrome [63]. Currently, there are two main therapeutic approaches to ALI/ARDS: supportive therapy and pharmacological interventions. Despite increased understanding of the pathophysiology of ARDS, the efficacy of preferred treatments such as lung-protective ventilation, prone positioning, and neuromuscular blockers is often limited [71]. Currently, there are no successful pharmacological therapies for ARDS [72]. T herefore, there is a need to study the effects of EVs on ALI/ARDS.

Extracellular vesicles are emerging as a promising therapeutic and diagnostic tool, with studies demonstrating their potential role in the treatment and diagnosis of digestive system diseases, cancer, and other areas of research [6, 73, 74]. Additionally, a meta-analysis have shown that stem cell-derived EVs improve cardiac function and reduce infarct size in myocardial infarction animals [75]. Another meta-analysis have found that MSC-EVs have therapeutic potential for acute and chronic liver diseases [76]. Furthermore, several meta-analysis studies have indicated that EVs are involved in the treatment of acute kidney injury and osteoporosis [77, 78]. However, some studies have suggested that EVs may not only have a therapeutic effect in disease development but may also contribute to disease progression.A study found that the increase of peripheral blood miR-1298-5p in sepsis-associated ALI patients triggered lung inflammation by inhibiting the proliferation of human bronchial epithelial cells and inducing epithelial permeability changes [79]. Another study revealed that EVs in the plasma of sepsis patients are rich in miR-210-3p, which can enhance various responses associated with sepsis-induced ALI by regulating autophagy and inflammation. These responses include macrophage inflammatory responses, bronchial epithelial cell apoptosis, and changes in lung microvascular endothelial cell permeability [80].

Recent studies have shown that there may be differences in the membrane lipid composition and capsule contents released by the same cells [81, 82]. In our meta-analysis, EVs of various sizes were included. the large heterogeneity among EVs is a major obstacle to understanding the composition and functional characteristics of the different secreted components. For further studies, the isolation, identification, and compositional analysis of EVs of different sizes are key determinants of ALI treatment. In addition, more efficient and safer methods of EVs preparation, isolation, characterisation and stockpiling skills are also important factors in determining whether EVs can be used on a large scale in the clinic.

### Limitations

Firstly, the overall sample size we analysed was very small due to the small sample size of the preclinical trial. Secondly, extracting data from drawings using Engauge Digitizer software in the absence of raw data may have altered the raw data and thus affected the results. This is despite the fact that we used a method where data were extracted separately by multiple reviewers. Finally, due to the lack of large animal tests and routine clinical parameters (e.g., respiratory mechanics), small tests may miss important information that is not conducive to guiding clinical applications.

## Conclusion

This meta-analysis demonstrated that stem cell-derived EV therapy could improve lung function and inflammatory response in preclinical ALI animal models, while non-stem cell-derived EVs aggravate lung injury. This result provides an essential clue for human clinical trials of EVs.

## Data Availability

The datasets used and/or analysed during the current study are available from the corresponding author on reasonable request.

## Abbreviations

Evs: Extracellular vesicles
ALI: Acute lung injury
ARDS: Acute respiratory distress syndrome
MSCs: Mesenchymal stem cells
ABs: Apoptotic bodies
MVs: Microvesicles
SMD: Standardised mean differences
BMSC: Bone marrow mesenchymal stromal cell
ADMSC: Adipose-derived mesenchymal stromal cell
WJ-MSC: Umbilical Wharton’s jelly mesenchymal stromal cell
AMs: Alveolar macrophages
UCMSC: Umbilical cord mesenchymal stromal cell
UVLC: Umbilical vein endothelial
EPC: Endothelial progenitor cells
LIS: Lung injury score
BLM: Bleomycin
LPS: Lipopolysaccharide
SwIV: Swine/MN/08
MDR-P: Multidrug-resistant PA

## References

[1] Shaw TD, McAuley DF, O'Kane CM (2019). Emerging drugs for treating the acute respiratory distress syndrome. Expert Opin Emerg Drugs.

[2] Khemani RG, Smith L, Lopez-Fernandez YM, Kwok J, Morzov R, Klein MJ, Yehya N, Willson D, Kneyber MCJ, Lillie J, Fernandez A, Newth CJL, Jouvet P, Thomas NJ (2019). Paediatric acute respiratory distress syndrome incidence and epidemiology (PARDIE): an international, observational study. Lancet Respir Med.

[3] Fan E, Brodie D, Slutsky AS (2018). Acute Respiratory Distress Syndrome: Advances in Diagnosis and Treatment. JAMA.

[4] Ortiz-Diaz E, Festic E, Gajic O, Levitt JE (2013). Emerging pharmacological therapies for prevention and early treatment of acute lung injury. Seminars in respiratory and critical care medicine.

[5] Impellizzeri D, Bruschetta G, Esposito E, Cuzzocrea S (2015). Emerging drugs for acute lung injury. Expert Opin Emerg Drugs.

[6] Xiang Z, Jiang B, Li W, Zhai G, Zhou H, Wang Y, Wu J (2022). The diagnostic and prognostic value of serum exosome-derived carbamoyl phosphate synthase 1 in HEV-related acute liver failure patients. J Med Virol.

[7] Xiang Z, Jiang C, Yang J, Huang L, Jiang B, Wang X, Gao C, Li M, Meng Y, Tong L, Ling B, Wang Y, Wu J (2023). Serum extracellular vesicle-derived ASS1 is a promising predictor for the occurrence of HEV-ALF. J Med Virol.

[8] Lee JW, Fang X, Gupta N, Serikov V, Matthay MA (2009). Allogeneic human mesenchymal stem cells for treatment of E. coli endotoxin-induced acute lung injury in the ex vivo perfused human lung. Proc Natl Acad Sci U S A.

[9] Lee C, Mitsialis SA, Aslam M, Vitali SH, Vergadi E, Konstantinou G, Sdrimas K, Fernandez-Gonzalez A, Kourembanas S (2012). Exosomes mediate the cytoprotective action of mesenchymal stromal cells on hypoxia-induced pulmonary hypertension. Circulation.

[10] Chen JY, An R, Liu ZJ, Wang JJ, Chen SZ, Hong MM, Liu JH, Xiao MY, Chen YF (2014). Therapeutic effects of mesenchymal stem cell-derived microvesicles on pulmonary arterial hypertension in rats. Acta Pharmacol Sin.

[11] Cruz FF, Borg ZD, Goodwin M, Sokocevic D, Wagner DE, Coffey A, Antunes M, Robinson KL, Mitsialis SA, Kourembanas S, Thane K, Hoffman AM, McKenna DH, Rocco PR, Weiss DJ (2015). Systemic Administration of Human Bone Marrow-Derived Mesenchymal Stromal Cell Extracellular Vesicles Ameliorates Aspergillus Hyphal Extract-Induced Allergic Airway Inflammation in Immunocompetent Mice. Stem Cells Transl Med.

[12] Lee JW, Krasnodembskaya A, McKenna DH, Song Y, Abbott J, Matthay MA (2013). Therapeutic effects of human mesenchymal stem cells in ex vivo human lungs injured with live bacteria. Am J Respir Crit Care Med.

[13] Zhu YG, Feng XM, Abbott J, Fang XH, Hao Q, Monsel A, Qu JM, Matthay MA, Lee JW (2014). Human mesenchymal stem cell microvesicles for treatment of Escherichia coli endotoxin-induced acute lung injury in mice. Stem cells (Dayton, Ohio).

[14] Monsel A, Zhu YG, Gennai S, Hao Q, Hu S, Rouby JJ, Rosenzwajg M, Matthay MA, Lee JW. Therapeutic Effects of Human Mesenchymal Stem Cell-derived Microvesicles in Severe Pneumonia in Mice. Am J Respir Crit Care Med. 2015;192(3):324–36. 10.1164/rccm.201410-1765OC. 10.1164/rccm.201410-1765OC PMC4584251 26067592

[15] Phinney DG, Di Giuseppe M, Njah J, Sala E, Shiva S, St Croix CM, Stolz DB, Watkins SC, Di YP, Leikauf GD, Kolls J, Riches DW, Deiuliis G, Kaminski N, Boregowda SV, McKenna DH, Ortiz LA (2015). Mesenchymal stem cells use extracellular vesicles to outsource mitophagy and shuttle microRNAs. Nat Commun.

[16] Gennai S, Monsel A, Hao Q, Park J, Matthay MA, Lee JW (2015). Microvesicles Derived From Human Mesenchymal Stem Cells Restore Alveolar Fluid Clearance in Human Lungs Rejected for Transplantation. Am J Transplant Off J Am Soc Transplant Am Soc Transplant Surg.

[17] Gunawardena TNA, Rahman MT, Abdullah BJJ, Abu Kasim NH (2019). Conditioned media derived from mesenchymal stem cell cultures: The next generation for regenerative medicine. J Tissue Eng Regen Med.

[18] Carnino JM, Lee H, Jin Y (2019). Isolation and characterization of extracellular vesicles from Broncho-alveolar lavage fluid: a review and comparison of different methods. Respir Res.

[19] Salimi L, Akbari A, Jabbari N, Mojarad B, Vahhabi A, Szafert S, Kalashani SA, Soraya H, Nawaz M, Rezaie J (2020). Synergies in exosomes and autophagy pathways for cellular homeostasis and metastasis of tumor cells. Cell Biosci.

[20] Babaei M, Rezaie J (2021). Application of stem cell-derived exosomes in ischemic diseases: opportunity and limitations. J Transl Med.

[21] Doyle LM, Wang MZ. Overview of Extracellular Vesicles, Their Origin, Composition, Purpose, and Methods for Exosome Isolation and Analysis. Cells. 2019;8(7). 10.3390/cells8070727. 10.3390/cells8070727 PMC6678302 31311206

[22] van Niel G, D'Angelo G, Raposo G (2018). Shedding light on the cell biology of extracellular vesicles. Nat Rev Mol Cell Biol.

[23] Xiang Z, Jiang C, Yang J, Huang L, Jiang B, Wang, X.,Wu, J.  (2023). Serum extracellular vesicle-derived ASS1 is a promising predictor for the occurrence of HEV-ALF. J Med Virol.

[24] Moher D, Liberati A, Tetzlaff J, Altman DG (2009). Preferred reporting items for systematic reviews and meta-analyses: the PRISMA statement. BMJ (Clinical research ed).

[25] Tierney JF, Stewart LA, Ghersi D, Burdett S, Sydes MR (2007). Practical methods for incorporating summary time-to-event data into meta-analysis. Trials.

[26] Wu XL, Tu Q, Faure G, Gallet P, Kohler C, Bittencourt Mde C (2016). Diagnostic and Prognostic Value of Circulating Tumor Cells in Head and Neck Squamous Cell Carcinoma: a systematic review and meta-analysis. Sci Rep.

[27] Macleod MR, O'Collins T, Howells DW, Donnan GA (2004). Pooling of animal experimental data reveals influence of study design and publication bias. Stroke.

[28] Higgins JP, Thompson SG, Deeks JJ, Altman DG (2003). Measuring inconsistency in meta-analyses. BMJ (Clinical research ed).

[29] Buesing KL, Densmore JC, Kaul S, Pritchard KA, Jr., Jarzembowski, J. A., Gourlay, D. M., Oldham, K. T.,  (2011). Endothelial Microparticles Induce Inflammation in Acute Lung Injury. J Surg Res.

[30] Chang C-L, Sung P-H, Chen K-H, Shao P-L, Yang C-C, Cheng B-C, Lin K-C, Chen C-H, Chai H-T, Chang H-W, Yip H-K, Chen H-H (2018). Adipose-derived mesenchymal stem cell-derived exosomes alleviate overwhelming systemic inflammatory reaction and organ damage and improve outcome in rat sepsis syndrome. American journal of translational research.

[31] Chen W, Wang S, Xiang H, Liu J, Zhang Y, Zhou S, Du T, Shan L (2019). Microvesicles derived from human Wharton's Jelly mesenchymal stem cells ameliorate acute lung injury partly mediated by hepatocyte growth factor. Int J Biochem Cell Biol.

[32] Chen WX, Zhou J, Zhou SS, Zhang YD, Ji TY, Zhang XL, Wang SM, Du T, Ding DG (2020). Microvesicles derived from human Wharton's jelly mesenchymal stem cells enhance autophagy and ameliorate acute lung injury via delivery of miR-100. Stem Cell Res Ther.

[33] Deng H, Wu L, Liu M, Zhu L, Chen Y, Zhou H, Shi X, Wei J, Zheng L, Hu X, Wang M, He Z, Lv X, Yang H (2020). Bone Marrow Mesenchymal Stem Cell-Derived Exosomes Attenuate LPS-Induced ARDS by Modulating Macrophage Polarization Through Inhibiting Glycolysis in Macrophages. Shock..

[34] Deng H, Zhu L, Zhang Y, Zheng L, Hu S, Zhou W, Zhang T, Xu W, Chen Y, Zhou H, Li Q, Wei J, Yang H, Lv X (2022). Differential Lung Protective Capacity of Exosomes Derived from Human Adipose Tissue, Bone Marrow, and Umbilical Cord Mesenchymal Stem Cells in Sepsis-Induced Acute Lung Injury. Oxid Med Cell Longev.

[35] Huang R, Qin C, Wang J, Hu Y, Zheng G, Qiu G, Ge M, Tao H, Shu Q, Xu J (2019). Differential effects of extracellular vesicles from aging and young mesenchymal stem cells in acute lung injury. Aging.

[36] Kaspi H, Semo J, Abramov N, Dekel C, Lindborg S, Kern R, Aricha R. MSC-NTF (NurOwn®) exosomes: a novel therapeutic modality in the mouse LPS-induced ARDS model. Stem Cell Res Ther. 2021;12(1):72. 10.1186/s13287-021-02143-w. 10.1186/s13287-021-02143-w PMC7814377 33468250

[37] Khatri M, Richardson LA, Meulia T (2018). Mesenchymal stem cell-derived extracellular vesicles attenuate influenza virus-induced acute lung injury in a pig model. Stem Cell Res Ther.

[38] Li Q-C, Jiang Y, Su Z-B (2019). Prophylactic treatment with MSC-derived exosomes attenuates traumatic acute lung injury in rats. American Journal of Physiology-Lung Cellular and Molecular Physiology.

[39] Liu F, Peng W, Chen J, Xu Z, Jiang R, Shao Q, Zhao N, Qian K (2021). Exosomes Derived From Alveolar Epithelial Cells Promote Alveolar Macrophage Activation Mediated by miR-92a-3p in Sepsis-Induced Acute Lung Injury. Front Cell Infect Microbiol.

[40] Liu J-H, Li C, Cao L, Zhang C-H, Zhang Z-H (2021). Exosomal miR-132-3p from mesenchymal stem cells alleviated LPS-induced acute lung injury by repressing TRAF6. Autoimmunity.

[41] Liu X, Gao C, Wang Y, Niu L, Jiang S, Pan S (2021). BMSC-Derived Exosomes Ameliorate LPS-Induced Acute Lung Injury by miR-384-5p-Controlled Alveolar Macrophage Autophagy. Oxid Med Cell Longev.

[42] Mao G-C, Gong C-C, Wang Z, Sun M-X, Pei Z-P, Meng W-Q, Cen J-F, He X-W, Lu Y, Xu Q-Q, Xiao K (2021). BMSC-derived exosomes ameliorate sulfur mustard-induced acute lung injury by regulating the GPRC5A-YAP axis. Acta Pharmacol Sin.

[43] Morrison TJ, Jackson MV, Cunningham EK, Kissenpfennig A, McAuley DF, O'Kane CM, Krasnodembskaya AD (2017). Mesenchymal Stromal Cells Modulate Macrophages in Clinically Relevant Lung Injury Models by Extracellular Vesicle Mitochondrial Transfer Am J Respir Crit Care Med..

[44] Shi MM, Zhu YG, Yan JY, Rouby JJ, Summah H, Monsel A, Qu JM. Role of miR-466 in mesenchymal stromal cell derived extracellular vesicles treating inoculation pneumonia caused by multidrug-resistant Pseudomonas aeruginosa. Clin Transl Med. 2021;11(1):e287. 10.1002/ctm2.287. 10.1002/ctm2.287 PMC7805403 33463070

[45] Silva JD, de Castro LL, Braga CL, Oliveira GP, Trivelin SA, Barbosa-Junior CM, Morales MM, Dos Santos CC, Weiss DJ, Lopes-Pacheco M, Cruz FF, Rocco PRM (2019). Mesenchymal Stromal Cells Are More Effective Than Their Extracellular Vesicles at Reducing Lung Injury Regardless of Acute Respiratory Distress Syndrome Etiology. Stem cells international.

[46] Dutra Silva J, Su Y, Calfee CS, Delucchi KL, Weiss D, McAuley DF, Krasnodembskaya AD. Mesenchymal stromal cell extracellular vesicles rescue mitochondrial dysfunction and improve barrier integrity in clinically relevant models of ARDS. Eur Respir J. 2021;58(1). 10.1183/13993003.02978-2020. 10.1183/13993003.02978-2020 PMC8318599 33334945

[47] Soni S, Wilson MR, O'Dea KP, Yoshida M, Katbeh U, Woods SJ, Takata M (2016). Alveolar macrophage-derived microvesicles mediate acute lung injury. Thorax.

[48] Tang X-D, Shi L, Monsel A, Li X-Y, Zhu H-L, Zhu Y-G, Qu J-M (2017). Mesenchymal Stem Cell Microvesicles Attenuate Acute Lung Injury in Mice Partly Mediated by Ang-1 mRNA. Stem cells (Dayton, Ohio).

[49] Varkouhi AK, Jerkic M, Ormesher L, Gagnon S, Goyal S, Rabani R, Masterson C, Spring C, Chen PZ, Gu FX, dos Santos CC, Curley GF, Laffey JG (2019). Extracellular Vesicles from Interferon-gamma-primed Human Umbilical Cord Mesenchymal Stromal Cells Reduce Escherichia coli-induced Acute Lung Injury in Rats. Anesthesiology.

[50] Wang J, Huang R, Xu Q, Zheng G, Qiu G, Ge M, Shu Q, Xu J (2020). Mesenchymal Stem Cell-Derived Extracellular Vesicles Alleviate Acute Lung Injury Via Transfer of miR-27a-3p*. Crit Care Med.

[51] Wu X, Liu Z, Hu L, Gu W, Zhu L (2018). Exosomes derived from endothelial progenitor cells ameliorate acute lung injury by transferring miR-126. Exp Cell Res.

[52] Xia L, Zhang C, Lv N, Liang Z, Ma T, Cheng H, Xia Y, Shi L (2022). AdMSC-derived exosomes alleviate acute lung injury via transferring mitochondrial component to improve homeostasis of alveolar macrophages. Theranostics.

[53] Xu J, Xu D, Yu Z, Fu Z, Lv Z, Meng L, Zhao X. Exosomal miR-150 partially attenuated acute lung injury by mediating microvascular endothelial cells and MAPK pathway. Biosci Rep. 2022;42(1). 10.1042/bsr20203363. 10.1042/BSR20203363 PMC8703023 34750610

[54] Xu N, Shao Y, Ye K, Qu Y, Memet O, He D, Shen J (2019). Mesenchymal stem cell-derived exosomes attenuate phosgene-induced acute lung injury in rats. Inhalation Toxicol.

[55] Xu X, Liu X, Dong X, Qiu H, Yang Y, Liu L (2022). Secretory Autophagosomes from Alveolar Macrophages Exacerbate Acute Respiratory Distress Syndrome by Releasing IL-1 beta. J Inflamm Res.

[56] Zhang L, Gao J, Qin C, Liang Y, Chen S, Hei F (2022). Inflammatory alveolar macrophage-derived microvesicles damage lung epithelial cells and induce lung injury. Immunol Lett.

[57] Zhao R, Wang L, Wang T, Xian P, Wang H, Long Q (2022). Inhalation of MSC-EVs is a noninvasive strategy for ameliorating acute lung injury. J Control Release.

[58] Smith KM, Mrozek JD, Simonton SC, Bing DR, Meyers PA, Connett JE, Mammel MC (1997). Prolonged partial liquid ventilation using conventional and high-frequency ventilatory techniques: gas exchange and lung pathology in an animal model of respiratory distress syndrome. Crit Care Med.

[59] Matute-Bello G, Downey G, Moore BB, Groshong SD, Matthay MA, Slutsky AS, Kuebler WM (2011). An official American Thoracic Society workshop report: features and measurements of experimental acute lung injury in animals. Am J Respir Cell Mol Biol.

[60] Wang F, Fang B, Qiang X, Shao J, Zhou L (2020). The efficacy of mesenchymal stromal cell-derived therapies for acute respiratory distress syndrome-a meta-analysis of preclinical trials. Respir Res.

[61] Nargesi AA, Lerman LO, Eirin A (2017). Mesenchymal Stem Cell-derived Extracellular Vesicles for Renal Repair. Curr Gene Ther.

[62] Grange C, Iampietro C, Bussolati B (2017). Stem cell extracellular vesicles and kidney injury. Stem Cell Investig.

[63] Sun X, Meng H, Wan W, Xie M, Wen C (2019). Application potential of stem/progenitor cell-derived extracellular vesicles in renal diseases. Stem Cell Res Ther.

[64] Eirin A, Zhu XY, Puranik AS, Woollard JR, Tang H, Dasari S, Lerman A, van Wijnen AJ, Lerman LO (2016). Comparative proteomic analysis of extracellular vesicles isolated from porcine adipose tissue-derived mesenchymal stem/stromal cells. Sci Rep.

[65] Anderson JD, Johansson HJ, Graham CS, Vesterlund M, Pham MT, Bramlett CS, Montgomery EN, Mellema MS, Bardini RL, Contreras Z, Hoon M, Bauer G, Fink KD, Fury B, Hendrix KJ, Chedin F, El-Andaloussi S, Hwang B, Mulligan MS, Lehtiö J, Nolta JA (2016). Comprehensive Proteomic Analysis of Mesenchymal Stem Cell Exosomes Reveals Modulation of Angiogenesis via Nuclear Factor-KappaB Signaling. Stem cells (Dayton, Ohio).

[66] Zhou Y, Li P, Goodwin AJ, Cook JA, Halushka PV, Chang E, Zingarelli B, Fan H (2019). Exosomes from endothelial progenitor cells improve outcomes of the lipopolysaccharide-induced acute lung injury. Critical care (London, England).

[67] Tkach M, Théry C (2016). Communication by Extracellular Vesicles: Where We Are and Where We Need to Go. Cell.

[68] Walker S, Busatto S, Pham A, Tian M, Suh A, Carson K, Quintero A, Lafrence M, Malik H, Santana MX, Wolfram J (2019). Extracellular vesicle-based drug delivery systems for cancer treatment. Theranostics.

[69] Valadi H, Ekström K, Bossios A, Sjöstrand M, Lee JJ, Lötvall JO (2007). Exosome-mediated transfer of mRNAs and microRNAs is a novel mechanism of genetic exchange between cells. Nat Cell Biol.

[70] Real JM, Ferreira LRP, Esteves GH, Koyama FC, Dias MVS, Bezerra-Neto JE, Cunha-Neto E, Machado FR, Salomão R, Azevedo LCP (2018). Exosomes from patients with septic shock convey miRNAs related to inflammation and cell cycle regulation: new signaling pathways in sepsis?. Critical care (London, England).

[71] Mokrá D (2020). Acute lung injury - from pathophysiology to treatment. Physiol Res.

[72] Lewis SR, Pritchard MW, Thomas CM, Smith AF (2019). Pharmacological agents for adults with acute respiratory distress syndrome. Cochrane database syst rev.

[73] Xiang Z, Li J, Zhang Z, Cen C, Chen W, Jiang B, Meng Y, Wang Y, Berglund B, Zhai G, Wu J (2022). Comprehensive Evaluation of Anti-PD-1, Anti-PD-L1, Anti-CTLA-4 and Their Combined Immunotherapy in Clinical Trials: A Systematic Review and Meta-analysis. Front Pharmacol.

[74] Wang J, Zhang P, Chen S, Duan H, Xie L, Yu J (2022). Microbiota and Gut Health: Promising Prospects for Clinical Trials from Bench to Bedside. Advanced Gut & Microbiome Research.

[75] Yang L, Zhu J, Zhang C, Wang J, Yue F, Jia X, Liu H (2019). Stem cell-derived extracellular vesicles for myocardial infarction: a meta-analysis of controlled animal studies. Aging.

[76] Fang X, Gao F, Yao Q, Xu H, Yu J, Cao H, Li S. Pooled Analysis of Mesenchymal Stromal Cell-Derived Extracellular Vesicle Therapy for Liver Disease in Preclinical Models. J Pers Med. 2023;13(3). 10.3390/jpm13030441. 10.3390/jpm13030441 PMC10056150 36983624

[77] Liu C, Wang J, Hu J, Fu B, Mao Z, Zhang H, Cai G, Chen X, Sun X (2020). Extracellular vesicles for acute kidney injury in preclinical rodent models: a meta-analysis. Stem Cell Res Ther.

[78] He X, Wang Y, Liu Z, Weng Y, Chen S, Pan Q, Li Y, Wang H, Lin S, Yu H (2023). Osteoporosis treatment using stem cell-derived exosomes: a systematic review and meta-analysis of preclinical studies. Stem Cell Res Ther.

[79] Ma J, Xu LY, Sun QH, Wan XY (2021). BingLi, Inhibition of miR-1298-5p attenuates sepsis lung injury by targeting SOCS6. Mol Cell Biochem.

[80] Gao M, Yu T, Liu D, Shi Y, Yang P, Zhang J, Wang J, Liu Y, Zhang X (2021). Sepsis plasma-derived exosomal miR-1–3p induces endothelial cell dysfunction by targeting SERP1. Clin sci..

[81] Karpman D, Ståhl AL, Arvidsson I (2017). Extracellular vesicles in renal disease. Nat Rev Nephrol.

[82] Lai RC, Tan SS, Yeo RW, Choo AB, Reiner AT, Su Y, Shen Y, Fu Z, Alexander L, Sze SK, Lim SK (2016). MSC secretes at least 3 EV types each with a unique permutation of membrane lipid, protein and RNA. Journal of extracellular vesicles.

